# Tubulogenesis of bovine uterine glands by epidermal growth factor and collagen I in 3D culture systems

**DOI:** 10.1042/BSR20260010

**Published:** 2026-06-08

**Authors:** Yosuke Sugino, Rei Ichikawa, Shuichi Matsuyama, Yuki Yamamoto, Kohei Kawano, Koji Kimura

**Affiliations:** 1 Graduate School of Environmental, Life, Natural Science and Technology, Okayama University, Okayama, Japan; 2 Graduate School of Bioagricultural Sciences, Nagoya University, Aichi, Japan; 3 Department of Veterinary Medicine, Tokyo University of Agriculture and Technology, Tokyo, Japan

**Keywords:** 3D cell culture, bovine, collagen I, epidermal growth factor, tubular structure, uterine gland

## Abstract

Uterine glands are endometrial exocrine epithelia that support early embryo development. Their secretions are particularly essential for conceptus elongation in cattle. Uterine glands develop from the luminal epithelium and elongate into the stromal layer toward the myometrium. This process is regulated by growth factors, WNT proteins, and the surrounding extracellular matrix (ECM); however, the precise mechanisms that govern bovine uterine gland morphogenesis remain unclear. In this study, we determined how these signaling factors and ECM components affect the tubular formation of bovine uterine gland fragments in 3D culture systems. Uterine gland fragments were enzymatically isolated from bovine endometria and 3D-cultured in Matrigel with or without growth factors (EGF, FGF1, FGF2, FGF7, FGF10, and IGF-1) and WNT (WNT3A, WNT5A, and WNT7A) proteins. Of these, only EGF stimulated the elongation of uterine gland fragments and eventually induced the formation of uterine gland-like structures. EGF-induced tubulogenesis was accompanied by a rapid increase in cell proliferation and alterations in cell–ECM interactions. The supplementation of collagen I with Matrigel further promoted the elongation of the tubular structures. Although the addition of collagen I did not alter the gene expression profiles of the uterine gland-like structures, the integrin-ROCK pathway contributed to the collagen-induced enhancement of elongation. Our findings clarified that EGF and collagen I, but not FGFs, IGF-1, or WNTs, are key regulators for the tubular formation of 3D-cultured bovine uterine gland fragments. This 3D culture system provides a new platform to examine the cellular and molecular mechanisms underlying bovine uterine gland morphogenesis.

## Introduction

Uterine glands are exocrine tubular epithelia located in the endometrium [1]. In response to the endocrine milieu, they secrete a variety of factors, including water, sugars, proteins, lipids, and ions, into the uterine lumen. These factors play important roles in supporting early embryonic development during peri-implantation [2]. In ruminants, the conceptus undergoes rapid elongation accompanied by trophoblast proliferation and a marked increase in interferon-tau secretion for maternal recognition of pregnancy [3–5]. Exposure of steroid hormones, such as progesterone or its analogs, to neonates of various species results in a partial or complete uterine gland-knockout (UGKO) phenotypes [6–9]. In partial UGKO heifers, the protein content of uterine luminal flushing is lower compared with that in untreated ones, suggesting that gland deficiency would affect the endometrial environment and reproductive outcome [6]. Complete UGKO ewes failed to support successful conceptus development because of the absence of uterine glands and their secretions [7]. Moreover, the complete UGKO mice also resulted in total infertility during adulthood [9]. These studies have strongly indicated the relationship between the secretory functions of uterine glands and reproductive outcome.

Uterine glands are derived from the luminal epithelium during the perinatal or neonatal period [7]. The luminal epithelia fold and infiltrate into the stromal layers, and the glandular epithelia subsequently elongate and branch toward the myometrium. In several species, signaling by growth factors (GFs) and WNT proteins (WNTs), as well as the composition of extracellular matrix (ECM) surrounding the uterine glands, have been implicated in neonatal gland morphogenesis [10]. After puberty, bovine uterine glands undergo cyclic proliferation and apoptosis, while overall gland morphology remains relatively constant [11]. In humans, uterine glands repeatedly degrade and reconstruct, depending on the menstrual cycle [12]. During gestation, uterine glands undergo hyperplasia and hypertrophy [13]. The mechanism governing gland morphogenesis remains incompletely understood. Direct real-time observations of uterine gland morphogenesis are technically challenging, as the glands are tightly surrounded by stromal cells and blood vessels within the endometrium. Moreover, the contribution of GFs and the ECM to gland morphogenesis is still poorly defined. Therefore, *in vitro* culture systems that can imitate gland tubulogenesis are needed.

To address this issue, three-dimensional (3D) culture systems have been adopted. Isolated uterine gland fragments from humans and mice are embedded into a basement membrane extract (e.g. Matrigel), typically form spherical structures (cysts). The cysts exhibit apico-basal polarity and maintain superior epithelial physiology compared with those established by conventional two-dimensional (2D) culture systems [14,15]. Bovine uterine gland fragments also form cysts and retain hormone responsiveness in 3D culture systems [16]. Nevertheless, these spherical structures do not recapitulate the native elongated tubular structures; thus, there are limitations to analyzing the tubulogenesis of uterine glands *in vitro*. Other tubular epithelia, such as mammary glands, salivary glands, and intestinal crypts, form tubular structures with elongation and branching in 3D culture systems under the influences of defined GFs, WNTs, and cell–ECM interactions [17–23]. Although studies have reported the formation of gland-like structures derived from human and mouse endometrial epithelia in culture systems [24–26], the regulatory mechanisms underlying this process are poorly understood.

In the present study, we examined 3D culture conditions that induce tubular structure formation from bovine uterine gland fragments and determined the contribution of GFs, WNTs, and ECMs to this process. This *in vitro* culture system provides a new platform for investigating the mechanisms underlying uterine gland morphogenesis.

## Materials and methods

### Preparation of bovine uteri

Bovine uteri without visible concepti were transported on ice from a local abattoir to the laboratory within 30–40 min following exsanguination. Macroscopic observation of the uteri and ovaries was performed to distinguish healthy uteri from those infected and to determine the estrous stage of the uteri, as previously described [27]. The stages were defined as follows: stage I (days 1–4 after ovulation), stage II (days 5–10), stage III (days 11–17), and stage IV (days 18–20). Uteri at Stage III with a corpus luteum on the ipsilateral ovary were selected for the experiments.

### Isolation of bovine uterine gland fragments

Bovine uterine gland fragments were enzymatically isolated as previously described [16]. Briefly, the stratum compactum and spongiosum of the endometrium were dissected and cut into 3–5-mm cubes. The tissues were digested in Hank’s balanced salt solution containing 165 units/ml collagenase (032-22364, Wako Pure Chemical Industries, Ltd., Osaka, Japan) and 200 units/ml DNase (DP, Worthington Biochemical Corp., NJ, USA) for 60–90 min at 38.5°C in a thermostatic water bath. The digested mixture was passed through a 150-μm mesh to remove undigested tissue. The filtrate was passed through a 70-μm mesh, which was inverted and rinsed with HBSS to collect uterine gland fragments trapped on the mesh, and transferred to a 50-ml centrifuge tube. The fragments were resuspended in phenol red-free DMEM/F12 (D2906, Thermo Fisher Scientific Inc., MA, USA) containing 0.1% bovine serum albumin (BSA, Nacalai Tesque, Inc., Kyoto, Japan). Using a stereomicroscope, 50 uterine gland fragments were picked and transferred to individual microtubes for subsequent experiments.

### Three-dimensional culture of bovine uterine gland fragments

The suspension containing uterine gland fragments was mixed with GF-reduced, phenol red-free Matrigel (356231, Corning Inc., NY, USA) at a 1:1 ratio (50%). The resulting mixture was seeded into 96-well plates (Thermo Fisher Scientific Inc.) and allowed to polymerize in a humidified incubator at 38.5°C for 30–60 min. After polymerization, culture medium consisted of phenol red-free DMEM/F12 supplemented with 0.1% BSA (Nacalai Tesque, Inc.), 10 ng/ml sodium selenite (S5621, Sigma–Aldrich Co. LLC, MO, USA), 4 μg/ml insulin (I6634, Sigma–Aldrich Co. LLC), 10 μg/ml transferrin (T101-5, BBI Solutions), 1 mM ascorbic acid (013-12061, Wako Pure Chemical Industries, Ltd.), and 20 μg/ml gentamycin (G1397, Sigma–Aldrich Co. LLC) was gently added onto the polymerized Matrigel suspension. The fragments were cultured for 5 days at 38.5°C in a humidified atmosphere containing 5% CO_2_ in air. The culture medium was replaced every 1–2 days.

### Time-lapse imaging of 3D-cultured bovine uterine gland fragments

A WSL-1800 CytoWatcher with an ImageSaverT (ATTO Co., Ltd., Tokyo, Japan) was used to obtain sequential images. The images were captured every 20 min for a total duration of 5 days. The temperature was maintained at 38.5°C in a humidified atmosphere containing 5% CO_2_ in air.

### Influence of growth factors and WNT proteins on the morphological changes in 3D-cultured bovine uterine gland fragments

According to previous studies, several GFs and WNT proteins contribute to tubular structure formation in mammary and salivary glands, as well as intestinal crypts *in vitro* [17,21,28]. We initially examined the effects of these factors on the morphological changes of 3D-cultured bovine uterine gland fragments. Culture media were supplemented with the following factors at various final concentrations: 1 or 5 ng/ml EGF (E9644, Sigma–Aldrich Co. LLC), 100 or 500 ng/ml FGF1 (064-04781, Wako Pure Chemical Industries, Ltd.), 100 or 200 ng/ml FGF2 (064-04541, Wako Pure Chemical Industries, Ltd.), 100 or 500 ng/ml FGF7 (119-00661, Wako Pure Chemical Industries, Ltd.), 100 or 1000 ng/ml FGF10 (060-04401, Wako Pure Chemical Industries, Ltd.), and 10, 50, or 100 ng/ml IGF-1 (4216, BioVision, Inc., MA, USA). FGFs were combined and used at either low (100 ng/ml each of FGF1, FGF2, FGF7, and FGF10) or high (500 ng/ml FGF1, 200 ng/ml FGF2, 500 ng/ml FGF7, and 1000 ng/ml FGF10) concentrations. We also evaluated the effects of WNT proteins on morphological changes in 3D-cultured bovine uterine gland fragments. The culture media were supplemented with WNT proteins at the following concentrations: 40 ng/ml WNT3A (NKMAX Co., Ltd., Gyeonggi, South Korea), 50 ng/ml WNT5A (645-WN-010, R&D Systems, Inc., MN, USA), and 50 ng/ml WNT7A (NBP2-22946, Novus Biologicals, LLC, CO, USA) with or without 5 ng/ml EGF (Sigma–Aldrich Co. LLC).

**Figure 1:**
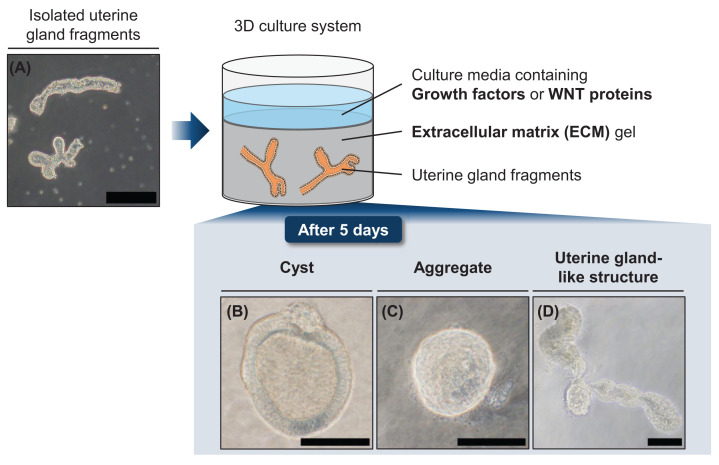
Schematic diagram representing the 3D culture system for bovine uterine gland fragments. Schematic diagram representing the 3D culture system for bovine uterine gland fragments. Isolated uterine gland fragments (A), cyst (B), aggregate (C), and UG-LIKE structure (D) are shown, respectively. Scale bars are 200 μm (A) and 100 μm (B–D).

Uterine gland fragments were cultured for 5 days at 38.5°C in a humidified atmosphere containing 5% CO_2_ in air. The culture medium was replaced every 1–2 days. Morphological changes in the 3D-cultured uterine gland fragments were observed using a digital camera (Digital Sight 1000, Nikon Corporation, Tokyo, Japan) or by phase contrast microscopy (FSX100; Olympus Corporation, Tokyo, Japan, or BZ-X800; Keyence Corporation, Osaka, Japan). The formation rates of cysts, aggregates, or uterine gland-like (UG-LIKE) structures (Figure 1) were determined as follows: the formation rates of cysts, aggregates, or UG-LIKE structures (%) = (number of cysts, aggregates, or UG-LIKE structures/total number of 3D-cultured uterine gland fragments) × 100.

### Cell proliferation in 3D-cultured bovine uterine gland fragments

To determine the extent of cell proliferation during morphological changes in 3D-cultured bovine uterine gland fragments, we evaluated the localization of Ki67, a proliferating cell marker, in 3D-cultured bovine uterine gland fragments. Uterine gland fragments were embedded in 50% Matrigel with or without 5 ng/ml EGF (Sigma–Aldrich Co. LLC) and harvested on days 1, 2, 3, and 5 of culture. Cysts and UG-LIKE structures were fixed in 4% paraformaldehyde in PBS for 15–30 min at room temperature (RT). Permeabilization was performed with 0.1% TritonX-100 in PBS for 10–15 min at RT. To reduce non-specific binding of the primary antibody, the cultured uterine glands were blocked in 5% BSA in PBS for more than 60 min at RT. Following blocking, glands were incubated overnight at RT with a primary antibody solution against Ki67 (1:100, M7240, Agilent, CA, USA). After washing with PBS, an incubation with secondary antibody [Goat anti-Mouse IgG (H + L) Cross-Adsorbed Secondary Antibody, Alexa Fluor 594, 1:500, A-11005, Thermo Fisher Scientific Inc.] solution was performed for 60 min at RT. The nuclei were counterstained with Hoechst 33342 (1:1000, H342, Dojindo, Tokyo, Japan). Images were acquired using a fluorescence microscope (BZ-X800, Keyence Corporation). The rate of Ki67-positive cells was calculated as follows: rate of Ki67-positive cells (%) = (number of Ki67-positive nuclei / Total number of nuclei) × 100.

### Cell polarity in 3D-cultured bovine uterine gland fragments

To examine the localization of cell polarity proteins in 3D-cultured bovine uterine gland fragments, we performed immunohistochemistry using an antibody against ZO-1. Experimental procedures, including fixation, permeabilization, blocking, and counterstaining were the same as described above. After blocking, cysts and UG-LIKE structures were incubated overnight at RT with the primary antibody solution against ZO-1 (1:100, sc-33725, Santa Cruz Biotechnology, Inc., TX, USA). After washing with PBS, secondary antibody [Goat anti-Rat IgG (H + L) Cross-Adsorbed Secondary Antibody, Alexa Fluor 594, 1:500, A-11007, Thermo Fisher Scientific Inc.] solution was added to the tissues and incubated for 60 min at RT. Images were acquired with a fluorescence microscope with sectioning module (BZ-X800, Keyence Corporation).

### RNA-seq analysis of uterine gland-like structures and cysts

To compare gene expression profiles between UG-LIKE structures and cysts, each sample was harvested from 3D culture systems with or without EGF, respectively (*N* = 3 per group). Total RNA was extracted using the RNeasy Plus Mini Kit (74134, Qiagen, Hilden, Germany) and stored at −80°C until subsequent processing. The RNA concentration and integrity were assessed using a 2100 Bioanalyzer (Agilent, CA, USA). Samples with an RNA integrity number >7.5 were used for RNA-seq analysis. cDNA libraries were prepared using the SMART-Seq HT PLUS Kit (Takara Bio, Shiga, Japan) and sequenced as paired-end 150 bp reads using a NovaSeq 6000 system (Illumina, CA, USA). After sequencing, adaptor sequences, low-quality sequences, and ambiguous nucleotides were trimmed. The filtered reads were aligned to the Bos taurus genome (ARS-UCD2.0) using CLC Genomics Workbench 25.0.1 (Qiagen) with default parameters. Gene expression was quantified as transcripts per million (TPM). Differentially expressed genes (DEGs) were determined based on a false discovery rate (FDR)-adjusted *P* < 0.05, Log_2_ (fold change, FC) >1 or <−1, and minimal TPM >1, followed by principal component analysis (PCA) and volcano plotting. Enriched pathways were identified using the Kyoto Encyclopedia of Genes and Genomes (KEGG) pathways under the database for annotation, visualization, and integrated discovery (DAVID) [29]. The RNA-sequencing data were deposited in the NCBI’s Gene Expression Omnibus (accession number: GSE315926) [30].

### Effects of collagen I supplementation on the morphological changes in 3D-cultured bovine uterine gland fragments

Although collagen I is one of ECM components within the bovine endometrium [31], its influence on uterine gland morphology remains poorly understood. To investigate the effect of collagen I on the morphological changes in 3D-cultured bovine uterine gland fragments, gland fragments were embedded in 50% Matrigel supplemented with collagen I.

Collagen I solution was prepared at 2 mg/ml following the manufacturer’s instructions (07001, Bovine Collagen I, STEMCELL Technologies Inc., BC, Canada). Uterine gland fragments were collected into individual microtubes and resuspended in phenol red-free DMEM/F12 (Thermo Fisher Scientific Inc.) containing 0.1% BSA (Nacalai Tesque, Inc.). The suspension was combined with GF-reduced, phenol red-free Matrigel (Corning Inc., NY, USA) and collagen I mixture. Final concentration of collagen I was 0, 0.1, and 0.5 mg/ml. The resulting mixtures were seeded into 96-well plates (Thermo Fisher Scientific Inc.) and polymerized in a humidified incubator at 38.5°C for 30–60 min. After polymerization, culture medium consisting of phenol red-free DMEM/F12 supplemented with 0.1% BSA (Nacalai Tesque, Inc.), 10 ng/ml sodium selenite (S5621, Sigma–Aldrich Co. LLC), 4 μg/ml insulin (I6634, Sigma–Aldrich Co. LLC), 10 μg/ml transferrin (T101-5, BBI Solutions), 1 mM ascorbic acid (013-12061, Wako Pure Chemical Industries, Ltd.), 20 μg/ml gentamycin (G1397, Sigma–Aldrich Co. LLC), and 5 ng/ml EGF (E9644, Sigma–Aldrich Co. LLC) was gently added onto the gels. The uterine gland fragments were cultured for 5 days at 38.5°C in a humidified atmosphere containing 5% CO_2_ in air. The culture medium was replaced every 1–2 days. Morphological changes in the 3D-cultured uterine gland fragments were observed and captured using a digital camera (Digital Sight 1000, Nikon Corporation, Tokyo, Japan) or by phase contrast microscope (FSX100; Olympus Corporation or BZ-X800; Keyence Corporation). The formation rates of cysts, aggregates, or UG-LIKE structures were determined as described above. Additionally, the length of the elongating parts of the UG-LIKE structures was measured using Fiji software [32].

To further assess how collagen I influences the morphological change of 3D-cultured uterine gland fragments, a potent α1β1 and α2β1 integrin inhibitor (TC-l 15; HY-107588, MedChemExpress LLC, NJ, USA) or ROCK inhibitor (Y-27632; HY-10583, MedChemExpress LLC) was added to the culture media. The gland fragments were cultured in Matrigel with or without 0.5 mg/ml collagen I for 5 days at 38.5°C in a humidified atmosphere containing 5% CO_2_ in air. The culture medium was replaced every 1–2 days. Observation and assessment of the morphological changes in 3D-cultured uterine gland fragments were performed as described above.

### RNA-seq analysis of uterine gland-like structures cultured in Matrigel with collagen I or Matrigel alone

Gene expression profiles in UG-LIKE structures were compared between Matrigel supplemented with or without 0.5 mg/ml collagen I (*N* = 3 per group). All analytical procedures were performed following as described above. The RNA-sequencing data were deposited in the NCBI’s Gene Expression Omnibus (accession number: GSE315926) [30].

### Statistical analyses

The experimental data are shown as the means ± standard error of the mean (SEM). Data normality and equality of variances were assessed using the Shapiro–Wilk and Brown–Forsythe tests, respectively. Parametric data were evaluated using a one-way or two-way analysis of variance (ANOVA), followed by Tukey’s multiple comparisons test. Non-parametric data were transformed according to their distribution prior to analysis: right-skewed continuous variables were log-transformed, whereas proportional data were arcsine square-root transformed. These resulting values were used for ANOVA and subsequent Tukey’s multiple comparisons test. Post hoc comparison was applied only when significant main effects or interactions were detected. All analyses were conducted using R [33]. *P*-values less than 0.05 were considered to be statistically significant.

## Results

### EGF induced the formation of uterine gland-like structures in 3D culture

**Figure 2:**
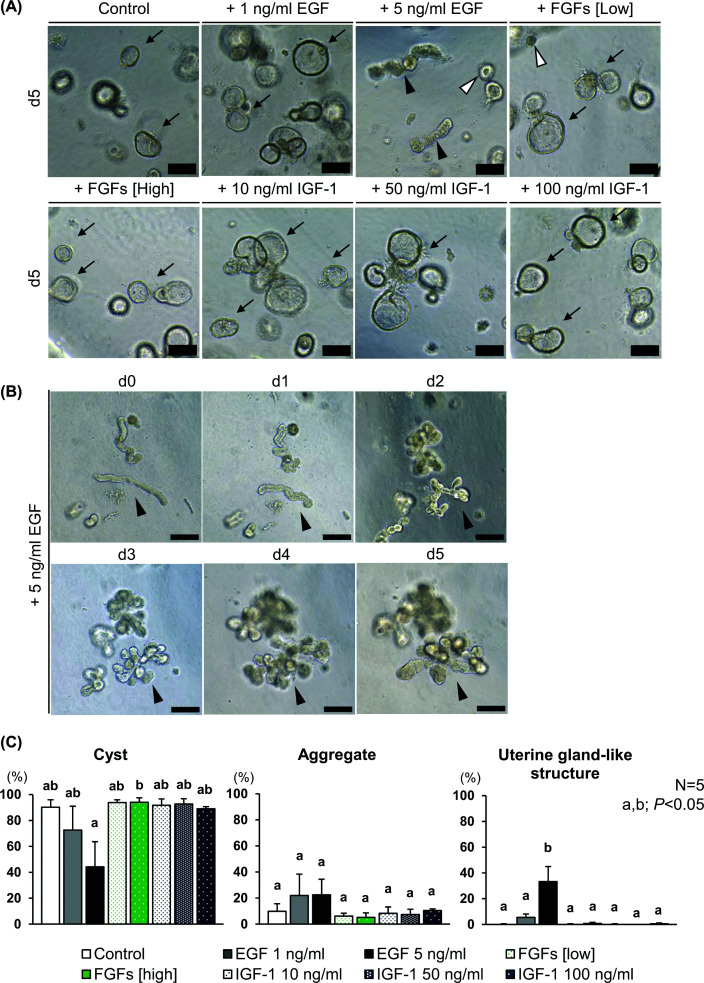
Effects of GFs on the morphological change of 3D-cultured bovine uterine glands. Effects of GFs on the morphological change of 3D-cultured bovine uterine glands. Microscopic images of bovine uterine glands after 5 days of 3D culture with or without EGF (1 or 5 ng/ml), Low or high FGFs (low: 100 ng/ml each of FGF1, FGF2, FGF7, and FGF10; high: 500 ng/ml FGF1, 200 ng/ml FGF2, 500 ng/ml FGF7, and 1000 g/ml FGF10), or IGF-1 (10, 50, or 100 ng/ml). Black arrows, white arrowheads, and black arrowheads indicate cysts, aggregates, and UG-LIKE structures, respectively. Scale bars are 200 μm (A). Sequential images of bovine uterine glands cultured in Matrigel with 5 ng/ml EGF for 5 days. Black arrowheads follow the formation of UG-LIKE structures. Scale bars are 200 μm (B). Formation rates of cysts, aggregates, and UG-LIKE structures after 5 days of culture (*N* = 5, mean ± SEM). Different superscripts indicate significant differences among each GF treatment, as determined using one-way ANOVA of arcsine-transformed data followed by Tukey’s multiple comparison test (*P* < 0.05) (C).

To investigate the effects of GFs on the morphology of 3D-cultured bovine uterine glands, isolated fragments of uterine gland were embedded in Matrigel and cultured for 5 days in the presence or absence of EGF, FGFs (FGF1, FGF2, FGF7, and FGF10), or IGF-1, respectively. Without GFs, uterine glands gradually expanded their luminal space and formed spherical cyst-like structures (Figure 2A, black arrows). Greater than 90% of the fragments formed cysts while the rest of the uterine glands formed aggregates (Figure 2C). In contrast, in the presence of 5 ng/ml EGF, uterine gland fragments formed elongated tubular structures resembling *in vivo* uterine glands (UG-LIKE structure) (Figure 2A, black arrowheads). These fragments underwent shrinkage and folding with twisting within the initial 1–2 days of culture, followed by elongation and branching over the culture period (Figure 2B, and other representative fragments are shown in Supplementary Video S1). However, at 1 ng/ml EGF, or in the presence of FGFs or IGF-1, the uterine gland fragments formed mainly cysts or aggregates, and failed to show UG-LIKE structures during the culture period (Figure 2A). The formation rate of UG-LIKE structures significantly increased in cultures containing 5 ng/ml EGF compared with all other conditions (Figure 2C).

### WNT proteins did not affect the formation of uterine gland-like structures

**Figure 3:**
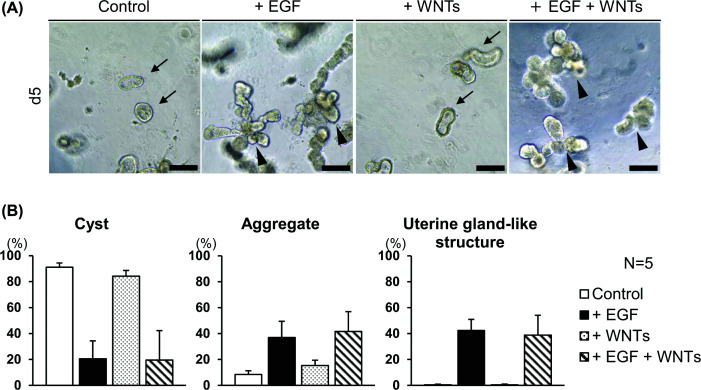
Effects of WNT proteins on the morphological change of 3D-cultured bovine uterine glands. Effects of WNT proteins on the morphological change of 3D-cultured bovine uterine glands. Microscopic images of bovine uterine glands after 5 days of 3D culture in the presence or absence of WNT proteins (40 ng/ml WNT3A, 50 ng/ml WNT5A, and 50 ng/ml WNT7A) and 5 ng/ml EGF. Black arrows and arrowheads indicate cysts and UG-LIKE structures, respectively. Scale bars are 200 μm (A). Formation rates of cysts, aggregates, and UG-LIKE structures after 5 days of culture (*N* = 5, mean ± SEM) (B).

To determine the effects of WNT proteins on the formation of UG-LIKE structures, uterine gland fragments were embedded in Matrigel and cultured for 5 days with or without WNT proteins (WNT3A, WNT5A, and WNT7A) and EGF, respectively. In the presence of WNT proteins without EGF, the fragments formed cysts during the culture period, similar to the control (Figure 3A, black arrows and Figure 3B). In contrast, co-treatment with WNT proteins and EGF induced the formation of UG-LIKE structures, which were comparable to those observed following EGF treatment (Figure 3A, black arrowheads and Figure 3B). A two-way ANOVA was performed on arcsine-transformed formation rates of cysts, aggregates, and UG-LIKE structures, with EGF and WNT protein treatments as two factors. EGF treatment had a significant effect on the formation rates of all structures (cysts and UG-LIKE structures: *P* < 0.001; aggregates: *P* < 0.01); however, neither WNT protein treatment nor interaction between EGF and WNT treatments had any significant effects on these rates (Supplementary Table S1).

### Polarity and proliferation of 3D-cultured bovine uterine glands

**Figure 4:**
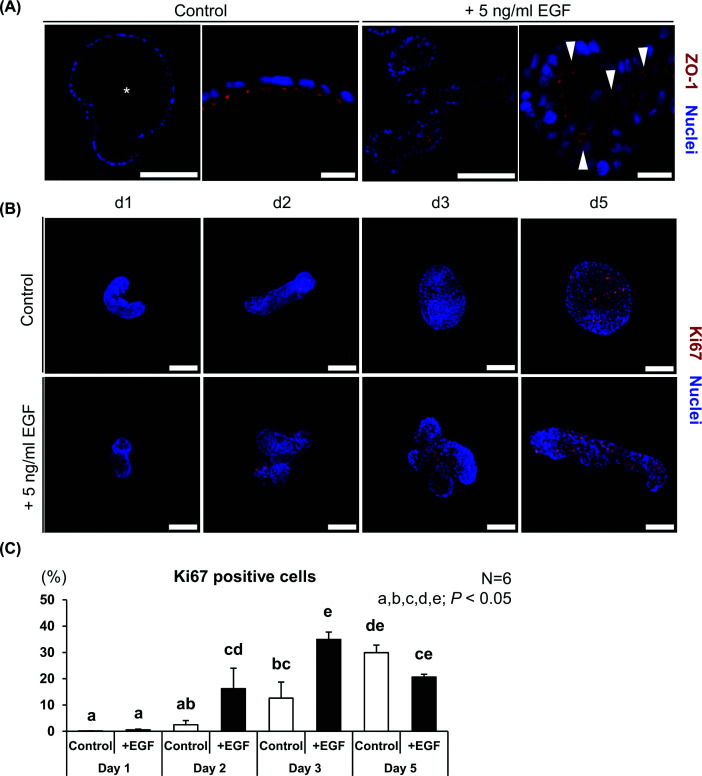
Cell polarity and proliferation in 3D-cultured bovine uterine glands. Cell polarity and proliferation in 3D-cultured bovine uterine glands. Immunohistochemical localization of tight junction marker (ZO-1) in 3D-cultured bovine uterine glands on day 5 with or without 5 ng/ml EGF. ZO-1: red and nuclei: blue. In each group, the right-side image shows a magnified view of the left-side image. An asterisk and white arrowheads indicate lumens. Scale bars in left- and right-side images represent 100 and 20 μm, respectively (A). Immunohistochemical localization of Ki67-staining cells in 3D-cultured bovine uterine glands on days 1, 2, 3, and 5, with or without 5 ng/ml EGF. Ki67: red and nuclei: blue. Scale bars represent 100 μm (B). Rate of Ki67-positive cells in 3D-cultured bovine uterine glands on days 1, 2, 3, and 5, with or without 5 ng/ml EGF (*N* = 6, mean ± SEM). Different superscripts indicate significant differences among each group, as determined using two-way ANOVA of arcsine-transformed data followed by Tukey’s multiple comparison test (*P* < 0.05) (C).

Localization of tight junction marker (ZO-1) in 3D-cultured bovine uterine glands was evaluated by immunohistochemistry (Figure 4A and Supplementary Figure S1A). ZO-1 was located along the luminal surface of both cysts (Control) and UG-LIKE structures (+5 ng/ml EGF). Cysts were characterized as a single continuous spherical lumen (Figure 4A, asterisk) and consisted of a single cell layer. In contrast, UG-LIKE structures showed multiple discontinuous luminal compartments (Figure 4A, white arrowheads), surrounded by stratified cell layers.

Cell proliferation in the 3D-cultured bovine uterine glands was evaluated by Ki67 immunostaining (Figure 4B,C, and Supplementary Figure S1B). On day 1, less than 1% of Ki67-positive cells were detected regardless of EGF treatment (control, 0.14% versus +5 ng/ml EGF, 0.55%). On days 2 and 3, EGF significantly increased the rate of Ki67-positive cells compared with the controls. Under EGF treatment, the proliferation rate was significantly higher on day 3 than on day 2 (day 2: control, 2.45% versus +5 ng/ml EGF, 16.2%; day 3: control, 12.6% versus +5 ng/ml EGF, 34.9%). However, the Ki67-positive cell rate in EGF treatment was comparable to that of the control on day 5 (control, 29.9% versus EGF, 20.6%), since the control group showed a gradual increase in proliferation from day 1 to day 5. The Ki67-positive cells were distributed throughout both cysts and UG-LIKE structures. A two-way ANOVA of arcsine-transformed Ki67-positive cell rates indicated the significant effects of EGF treatment (*P* < 0.01), culture period (*P* < 0.001), and their interaction (*P* < 0.01) (Supplementary Table S2).

### Comparison of gene expression patterns between uterine gland-like structures and cysts

**Figure 5:**
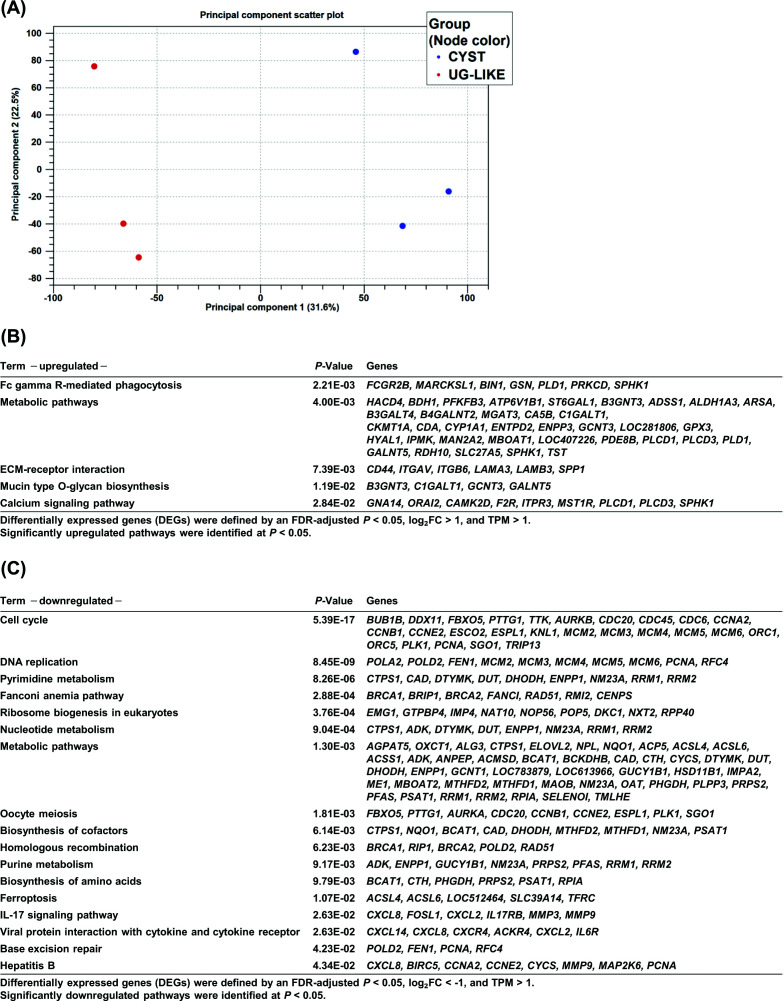
RNA-seq analysis comparing 3D-cultured bovine uterine glands with or without EGF treatment (UG-LIKE structures versus cysts). RNA-seq analysis comparing 3D-cultured bovine uterine glands with or without EGF treatment (UG-LIKE structures versus cysts). PCA plot of transcriptomic data in cysts (CYST, without EGF) and UG-LIKE structures (UG-LIKE, with EGF) on day 5 (*N* = 3 per group) (A). Upregulated enriched KEGG pathways identified from DEGs in UG-LIKE structures compared with cysts using an FDR-adjusted *P* < 0.05, Log_2_(fold change, FC) >1, and minimum TPM >1 (*N* = 3 per group) (B). Downregulated enriched KEGG pathways identified from DEGs in UG-LIKE structures compared with cysts using an FDR-adjusted *P* < 0.05, Log_2_(FC) < −1, and TPM > 1 (*N* = 3 per group) (C).

As shown in Figure 2, uterine gland fragments were transformed into UG-LIKE structures in the presence of EGF, whereas cysts, in its absence. To examine transcriptional differences between these two structures, RNA-seq analysis was performed on samples collected on day 5. PCA revealed a clear separation between the UG-LIKE structures and cysts (CYST) groups, with the first two principal components explaining 31.6% and 22.5% of the variance, respectively (Figure 5A). Hierarchical clustering dendrogram further confirmed that samples from CYST and UG-LIKE groups clustered into two distinct branches, indicating markedly different transcriptomic profiles (Supplementary Figure S2A). Differential expression analysis identified 593 DEGs in the UG-LIKE group compared with the CYST group, comprising 279 upregulated genes and 314 downregulated genes [Supplementary Figure S2B and Supplementary Table S3; an FDR-adjusted *P* < 0.05, Log_2_ (fold change, FC) >1 or <−1, and minimum TPM >1]. KEGG pathway analysis of the DEGs in the UG-LIKE group showed the upregulated and downregulated enriched pathways (Figure 5B,C), respectively. Genes associated with Fc gamma R-mediated phagocytosis, Metabolic pathways, ECM–receptor interaction, Mucin type O-glycan biosynthesis, and Calcium signaling pathway were upregulated (Figure 5B, *P* < 0.05). By contrast, genes involved in cell cycle, DNA replication, pyrimidine metabolism, fanconi anemia pathway, ribosome biogenesis in eukaryotes, nucleotide metabolism, metabolic pathways, oocyte meiosis, biosynthesis of cofactors, homologous recombination, purine metabolism, biosynthesis of amino acids, ferroptosis, IL-17 signaling pathway, viral protein interaction with cytokine and cytokine receptor, base excision repair, and hepatitis B were downregulated (Figure 5C, *P* < 0.05).

### Collagen I supplementation promoted the elongation of uterine gland-like structures

**Figure 6:**
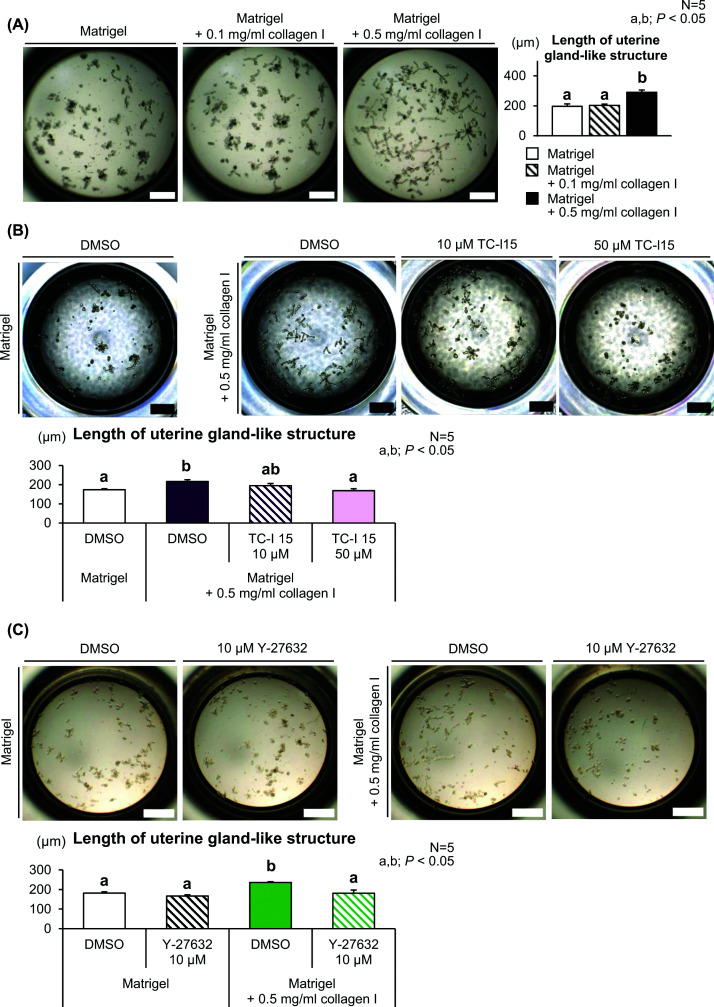
Morphological changes of UG-LIKE structures in collagen I-supplemented Matrigel, with or without the allosteric collagen I-binding integrin inhibitor and ROCK inhibitor. Morphological changes of UG-LIKE structures in collagen I-supplemented Matrigel, with or without the allosteric collagen I-binding integrin inhibitor and ROCK inhibitor. Lengths of UG-LIKE structures on day 5 in Matrigel supplemented with 0, 0.1, or 0.5 mg/ml bovine collagen I (*N* = 5, mean ± SEM). Different superscripts indicate significant differences among collagen I concentrations, as determined by one-way ANOVA of logit-transformed data (*P* < 0.05). Scale bars represent 1000 μm (A). Lengths of UG-LIKE structures on day 5 in Matrigel supplemented with 0 or 0.5 mg/ml bovine collagen I, with or without 0, 10, or 50 μM allosteric collagen I-binding integrin inhibitor (TC-l 15) (*N* = 5, mean ± SEM). Different superscripts indicate significant differences among experimental groups, combining collagen I supplementation and TC-l 15 treatment as a single factor, as determined by one-way ANOVA of logit-transformed data (*P* < 0.05). Scale bars represent 1000 μm (B). Lengths of UG-LIKE structures on day 5 in Matrigel supplemented with 0 or 0.5 mg/ml bovine collagen I, in the presence or absence of 0 or 10 μM ROCK inhibitor (Y-27632) (*N* = 5, mean ± SEM). Different superscripts indicate significant differences among experimental groups, with collagen I supplementation and Y-27632 treatment as two independent factors, as determined by two-way ANOVA of logit-transformed data (Supplementary Table S4, *P* < 0.05). Scale bars represent 1000 μm (C).

Although Matrigel primarily consists of basement membrane ECM components, such as laminin, collagen IV, nidogen, and entactin, the endometrium also contains other ECM proteins, including collagen I [34]. To investigate the effects of ECM components on the morphological changes occurring in 3D-cultured bovine uterine glands, fragments of uterine glands were cultured in Matrigel supplemented with 0, 0.1, 0.5 mg/ml bovine collagen I. The supplementation with 0.5 mg/ml collagen I significantly increased the length of UG-LIKE structures compared with 0 and 0.1 mg/ml collagen I (Figure 6A). In contrast, collagen I supplementation did not affect the formation rates of cysts, aggregates, or UG-LIKE structures (Supplementary Figure S3A).

To examine how collagen I influences the morphological change of UG-LIKE structures, inhibitors involved in the recognition of collagen I and regulation of the cytoskeleton were added into the culture system described above. The addition of 50 μM allosteric collagen I-binding integrin inhibitor (TC-l 15) significantly suppressed the collagen I-induced elongation of UG-LIKE structures (Figure 6B). Similarly, the addition of 10 μM ROCK inhibitor (Y-27632) significantly attenuated the elongation of UG-LIKE structures by collagen I, but not in Matrigel alone (Figure 6C and Supplementary Table S4). In contrast, neither inhibitor altered the formation rates of cysts, aggregates, and UG-LIKE structures (Supplementary Figure S6B,C, and Supplementary Table S4).

RNA-seq analysis was performed on UG-LIKE structures cultured for 5 days in Matrigel supplemented with 0.5 mg/ml collagen I (MAT + COL) or in Matrigel alone (MAT). PCA plot showed no clear separation between the MAT + COL and MAT groups, with the first two principal components explaining 37.3% and 26.7% of the variance (Supplementary Figure S4A). Consistently, the hierarchical clustering dendrogram divided each sample primarily by individuals rather than by collagen I supplementation (Supplementary Figure S4B). Differential expression analysis did not identify significant DEGs between the MAT + COL and MAT groups (Supplementary Figure S4C; an FDR-adjusted *P* < 0.05, Log_2_ FC >1 or <−1, and TPM >1).

## Discussion

Although GFs, WNT signaling, and cell–ECM interactions are known to be involved in uterine gland formation *in vivo* [2,10,13], the underlying mechanisms governing this process remain unclear. Real-time observation of gland morphogenesis *in vivo* is technically difficult because uterine glands are deeply embedded within stromal cells, blood vessels, and the surrounding ECM [10,31]. Therefore, an *in vitro* culture system for uterine glands is necessary to directly investigate glandular tubulogenesis. In this study, we determined how GFs, WNT proteins, and ECM components regulate the morphology of 3D-cultured bovine uterine gland fragments.

During EGF stimulation, 3D-cultured bovine uterine gland fragments formed tubular UG-LIKE structures (Figure 2). These marked morphological changes likely arise from the interplay between proliferating cells and the surrounding ECM, which provides mechanical resistance at the tissue-matrix interface. To enlarge the tissue volume within the ECM, tissues need to generate outward forces against it by increasing proliferation and internal cell density [20,35]. Consistent with this issue, EGF-treated uterine gland fragments showed undulation, elongation, and stratification from days 1 to 3 (Figures 2B and 4A,B). EGF also rapidly increased the rate of Ki67-positive cells in 3D-cultured uterine gland fragments on days 2 and 3 compared with the control condition (Figure 4C). In contrast, uterine gland fragments cultured under control conditions expanded their lumina and eventually formed spherical cysts rather than tubular structures (Figure 4A,B), even though their Ki67-positive cells gradually increased by day 5 to levels comparable to those in the EGF-treated group (Figure 4C). Lumen expansion elevates intraluminal pressure and stimulates cell proliferation. Elevated intraluminal pressure also enhances mechanical tension and maintains epithelial acini [36,37]. Similarly, in our present results, pathways related to cell proliferation, such as cell cycle and DNA replication, were upregulated in cysts relative to UG-LIKE structures on day 5 (Figure 5C). These may imply that the rapid increase in proliferative activity could elevate internal cell density and generate outward forces that promote the elongation of uterine gland fragments, whereas increased intraluminal pressure in control cysts could stimulate cell proliferation and stabilize cell–cell connections as well, thereby suppressing the budding and elongation as observed for UG-LIKE structure formation. In addition, remodeling of the surrounding ECM is also involved in the elongation of epithelial tissues [17,18]. As shown in Figure 5B, EGF significantly upregulated genes associated with the ECM–receptor interaction pathway, including *CD44*, *ITGAV*, *ITGB6*, *LAMA3*, *LAMB3*, and *SPP1*, in 3D-cultured uterine gland fragments. For example, EGFR signaling cooperates with CD44 to regulate invasive behavior in breast epithelial cells [38]. This finding raises the possibility that interactions between EGF signaling and these ECM–receptor molecules could be involved in uterine gland morphogenesis.

In contrast to EGF, other GFs, including FGFs and IGF1, did not induce the formation of UG-LIKE structures (Figure 2A). In lung and kidney epithelial cells, EGF induces elongation and branching [39,40], whereas FGFs regulate the morphogenesis of mammary and salivary glands [19,21,41]. IGF1 is also involved in duct and branch formation in mammary glands [42,43]. These observations suggest that the dependence of tubular structure formation on GFs is highly tissue-specific. One possible explanation for this is the context-dependent interaction between cells and ECMs. In 3D culture, the mechanical property of ECM regulates integrin-FAK/Src pathways, which in turn modulate GF receptor activity and downstream signaling, such as MAPK [44]. This may determine cellular responsiveness to specific GFs in the current study. Future studies elucidate ECM-integrin-GF receptor crosstalk in uterine gland morphogenesis.

Over the past few years, the existence of a stem cell niche in the bovine uterine glands has been investigated [45]. Stem cell niche contains Wnt-dependent stem/progenitor cells, such as Lgr5-expressing cells, which contribute to the maintenance of tissue morphology by regulating cell proliferation and differentiation [46]. It is well known that gastric glands and intestinal crypts have Lgr5-expressing cells *in vivo* [47], and organoids derived from these tissues form bud-like structures in response to Wnt signaling [28,48,49]. Similarly, Lgr5-positive cells have also been identified in the mouse and human uterine glands. These cells are considered to be involved in neonatal development and cyclic regeneration of uterine glands [50,51]. By contrast, in the bovine endometrium, stem-like side-population cells are identified only in the stromal layers, but not in the uterine glands [52]. In line with this observation, the formation of bovine UG-LIKE structures did not respond to WNTs addition (Figure 3). Taken together, these findings suggest that adult bovine uterine glands are unlikely to contain Wnt-dependent stem/progenitor cells, at least under our culture conditions. This may be reasonable because, unlike the adult primate endometrium, the morphology of the bovine endometrium remains relatively constant throughout the estrous cycle [53].

ECM components are known to affect tissue morphogenesis. Previous studies have shown that the elongation of 3D-cultured mammary glands is promoted by increasing collagen I density in mixed Matrigel [54]. The present study showed that supplementation of collagen I with Matrigel promoted the elongation of UG-LIKE structures compared with Matrigel alone. However, the collagen I-induced increase in elongation of UG-LIKE structures appeared to rely primarily on mechanical signaling through the integrin-ROCK pathway, rather than on transcriptional regulation (Figure 6 and Supplementary Figure S4). Similar phenomena have been reported in mammary epithelial cells. ROCK-mediated cellular contractility regulates the morphogenesis of 3D-cultured breast epithelial cells in collagen I matrix [55], and mammary epithelial structures elongate along collagen I fiber orientations in a Rac1- and ROCK-dependent manner [56]. It is well known that Rac and Rho GTPases regulate the actin cytoskeleton and cell motility through protein–protein interactions [57]. It is possible that uterine gland elongation is influenced by the surrounding collagen I matrix through modulation of cytoskeletal tension at the mechanical level. Their precise interaction should be further investigated.

In summary, we investigated the effects of GFs, WNTs, and ECM components on the formation of UG-LIKE structures. Our newly established 3D culture system of bovine uterine gland fragments would provide a valuable platform for further studying gene expression profiles and mechanical properties that govern the gland morphogenesis. It will also facilitate studies on the secretory functions of UG-LIKE structures in response to hormonal stimulation.

## Supplementary Material

Supplementary Figures S1-S4 and Tables S1-S4

Supplementary Figures Video S1

Supplementary Video S1

## Data Availability

The data supporting the findings of this study are available in the article and its supplemental figures, tables, and a video. The RNA-seq data discussed in this publication have been deposited in NCBI’s Gene Expression Omnibus [58] and are accessible through GEO Series accession number GSE315926 (https://www.ncbi.nlm.nih.gov/geo/query/acc.cgi?acc=GSE315926) [30].

## Abbreviations

ANOVA: analysis of variance
BSA: bovine serum albumin
DEG: Differentially expressed gene
ECM: extracellular matrix
FDR: false discovery rate
GF: growth factor
HBSS: Hanks' Balanced Salt Solution
KEGG: Kyoto Encyclopedia of Genes and Genomes
PCA: principal component analysis
RT: room temperature
SEM: standard error of the mean
TPM: transcripts per million
UGKO: uterine gland-knockout
UG-LIKE: uterine gland-like
