# Supplementary material for: Tubulogenesis of bovine uterine glands by epidermal growth factor and collagen I in 3D culture systems
Source: Biosci Rep. 2026 Jun 8;46(6):BSR20260010. doi: 10.1042/BSR20260010 (PMC13259824; doi:10.1042/BSR20260010)
Supplement: Supplementary Figures S1-S4 and Tables S1-S4 [file BSR-2026-0010_supp.pdf]

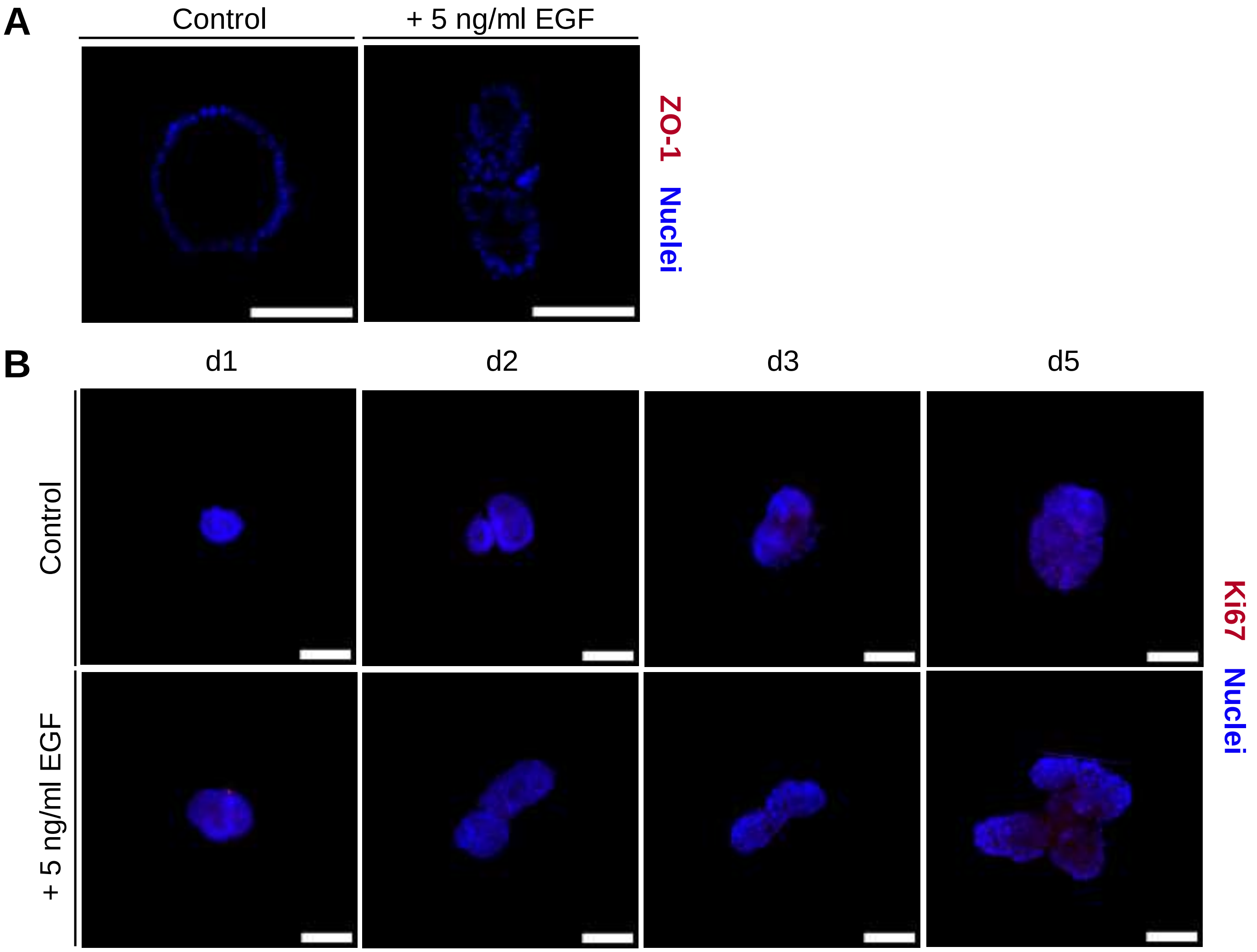

Figure S1

Negative controls of immunohistochemistry. (A) Negative control images of ZO-1 immunostaining in 3D-cultured bovine uterine glands on day 5 with or without 5 ng/ml EGF. ZO-1: red and nuclei: blue. Scale bars represent 100 μm. (B) Negative control images of Ki67 immunostaining in 3D-cultured bovine uterine glands on days 1, 2, 3, and 5, with or without 5 ng/ml EGF. Ki67: red and nuclei: blue. Scale bars represent 100 μm.

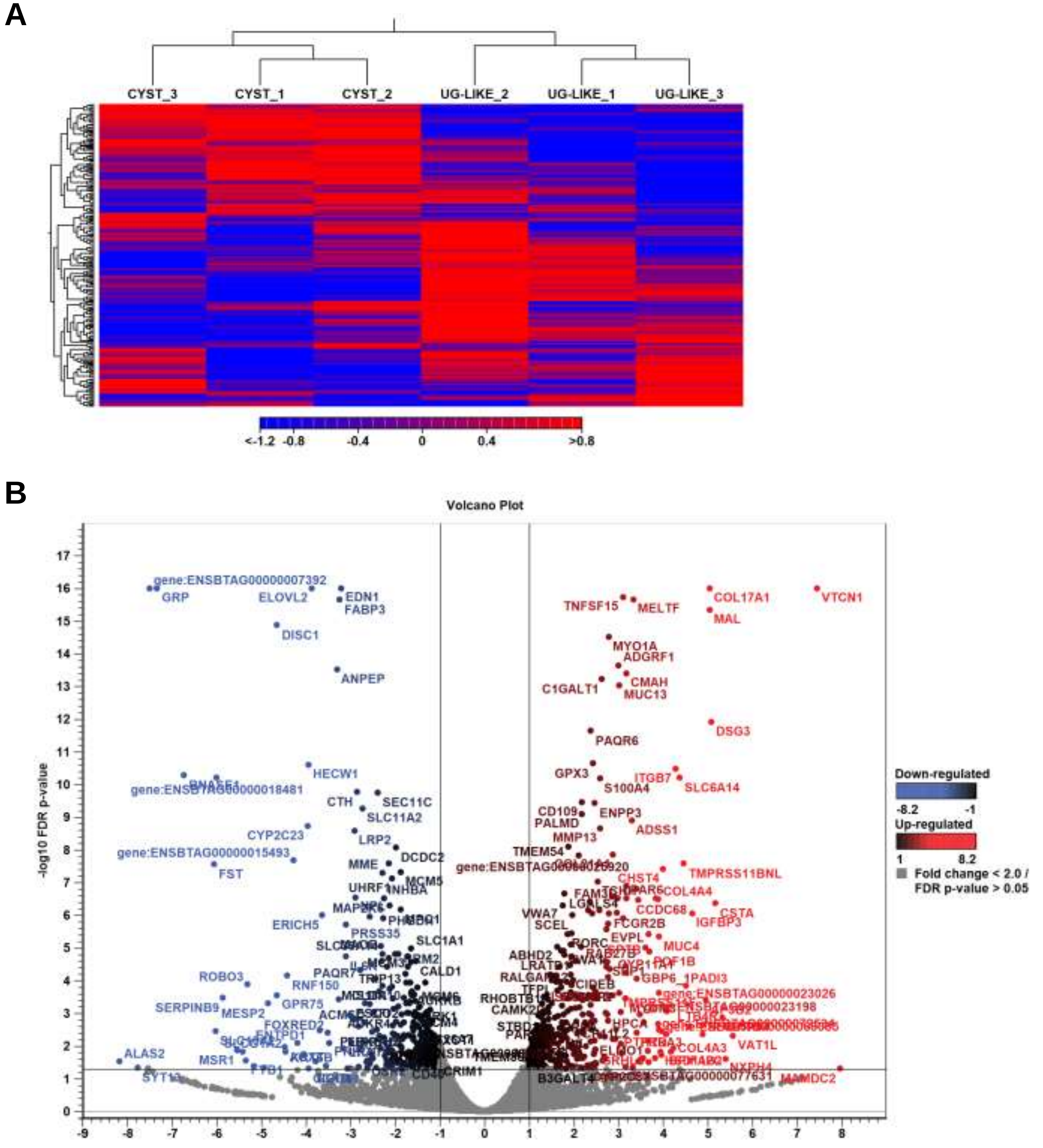

Figure S2

Cluster analysis and volcano plot of transcriptomic data in cysts and uterine gland-like structures. (A) Hierarchical cluster analysis and heatmap of the top 2,000 most variable genes across all samples (CYST_1, 2, and 3; UG-LIKE_1, 2, and 3). Distances were calculated using Euclidean distance and clustered with complete linkage. Dendrograms display the clustering of both genes (rows) and samples (columns). Red: upregulated and blue: downregulated. (B) Volcano plot of transcriptomic data in uterine gland-like structures compared with cysts on day 5. Red: upregulated and blue: downregulated. DEGs were identified using an FDR-adjusted $P < 0.05$, $\mathrm{Log_2FC} > 1$ or < -1, and minimum TPM > 1 (N = 3 per group).

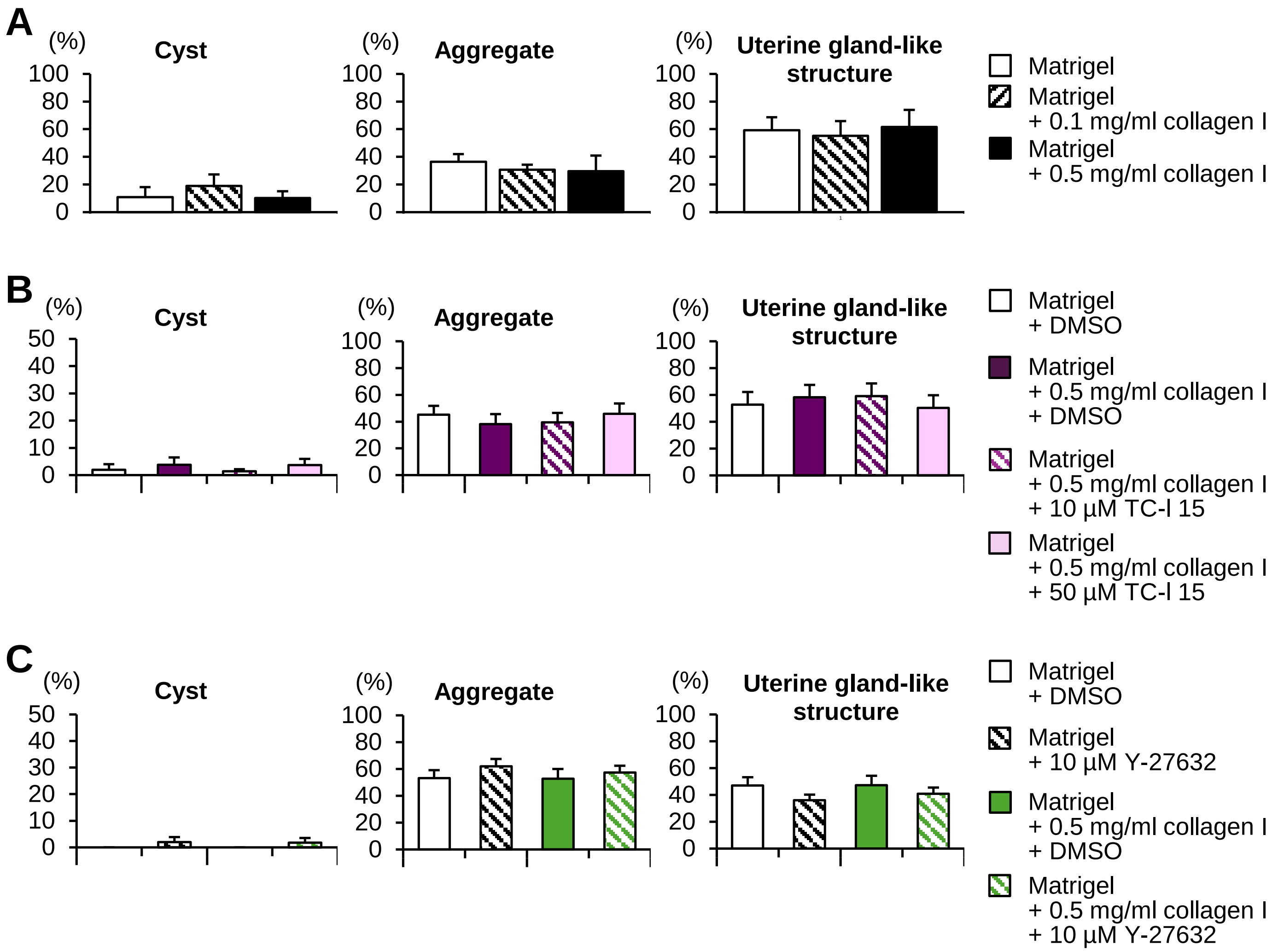

Figure S3

Formation rates of cysts, aggregates, and uterine gland-like structures in collagen I-supplemented Matrigel, with or without the allosteric collagen I-binding integrin inhibitor and ROCK inhibitor. (A) Formation rates of cysts, aggregates, and uterine gland-like structures on day 5 in Matrigel supplemented with 0, 0.1, or 0.5 mg/ml bovine collagen I (N = 5, mean ± SEM). Statistical differences were determined by one-way ANOVA of arcsine-transformed data, comparing collagen I concentrations ($P < 0.05$). (B) Formation rates of cysts, aggregates, and uterine gland-like structures on day 5 in Matrigel supplemented with 0 or 0.5 mg/ml bovine collagen I, with or without 0, 10, or 50 µM allosteric collagen I-binding integrin inhibitor (TC-l 15) (N = 5, mean ± SEM). Statistical differences were determined by one-way ANOVA of arcsine-transformed data, comparing experimental groups with collagen I supplementation and TC-l 15 treatment as a single factor ($P < 0.05$). (C) Formation rates of cysts, aggregates, and uterine gland-like structures on day 5 in Matrigel supplemented with 0 or 0.5 mg/ml bovine collagen I, with or without 0 or 10 µM ROCK inhibitor (Y-27632) (N = 5, mean ± SEM). Statistical differences were determined by two-way ANOVA of arcsine-transformed data, comparing experimental groups with collagen I supplementation and Y-27632 treatment as two independent factors ($P < 0.05$).

A

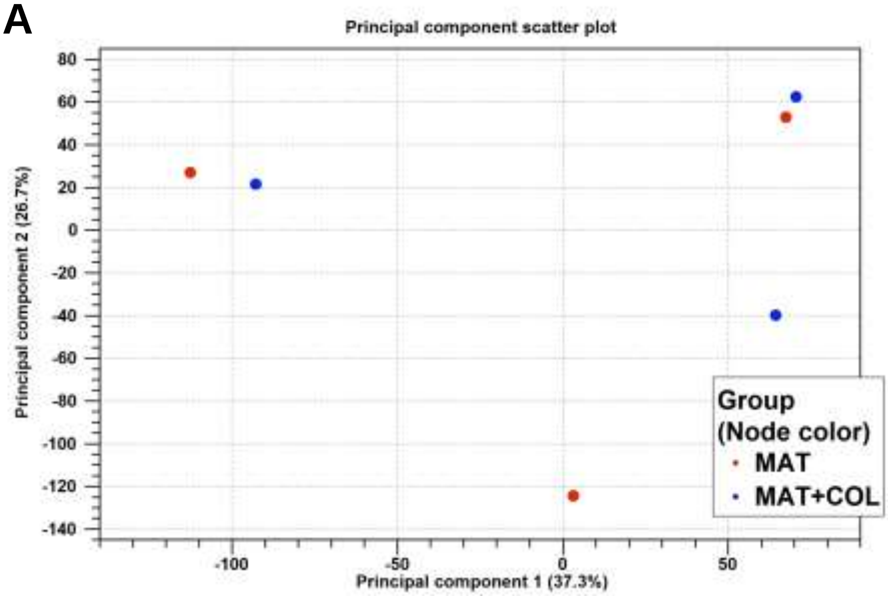
Principal component scatter plot
Principal component 2 (26.7%)
Principal component 1 (37.3%)
80
60
40
20
0
-20
-40
-60
-80
-100
-120
-140
-100
-50
0
50
Group
(Node color)
MAT
MAT+COL

B

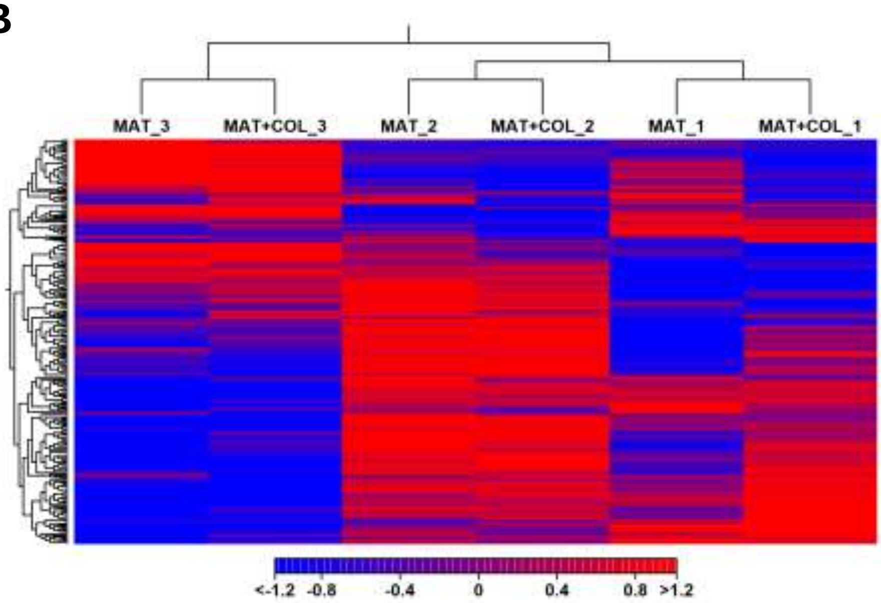
MAT_3
MAT+COL_3
MAT_2
MAT+COL_2
MAT_1
MAT+COL_1
<-1.2 -0.8 -0.4 0 0.4 0.8 >1.2

C

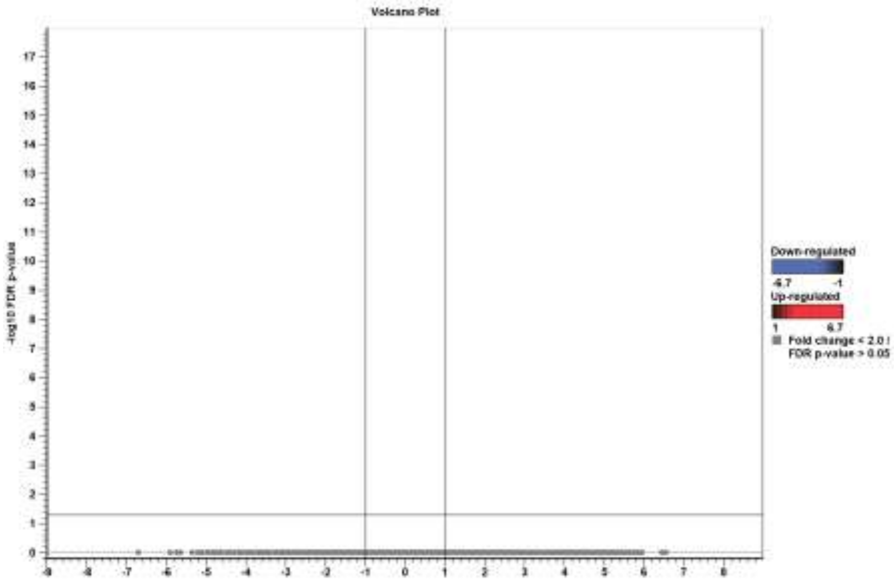
Volcano Plot
-log10 FDR p-value
Down-regulated
-6.7
-1
Up-regulated
1
6.7
Fold change < 2.0 /
FDR p-value > 0.05

Figure S4

RNA-seq analysis comparing uterine gland-like structures between in collagen I-supplemented Matrigel and Matrigel alone. (A) Principal component analysis (PCA) plot of transcriptomic data in uterine gland-like structures cultured in Matrigel alone (MAT) or in Matrigel supplemented with 0.5 mg/ml collagen I (MAT + COL) for 5 days (N = 3 per group). (B) Hierarchical cluster analysis and heatmap of the top 2,000 most variable genes among all samples (MAT_1, 2, and 3; MAT + COL_1, 2, and 3). Distances were calculated using Euclidean distance and clustered with complete linkage. Dendrograms display the clustering of both genes (rows) and samples (columns). Red: upregulated and blue: downregulated. (C) Volcano plot of transcriptomic data in uterine gland-like structures cultured in MAT or in MAT + COL for 5 days. Red: upregulated and blue: downregulated. DEGs were identified using an FDR-adjusted $P < 0.05$, $Log_2FC > 1$ or < -1, and TPM > 1 (N = 3 per group).

Table S1

Table of two-way ANOVA performed on arcsine-transformed arcsine- transformed formation rate of cysts, aggregates, and uterine gland-like structures, with EGF and WNT treatments as two factors.

	F-value		
	**Cyst**	**Aggregate**	**Uterine gland-like structure**
**EGF**	36.28 ***	10.84 **	31.99 ***
**WNT**	0.13	0.48	0.06
**EGF × WNT**	0.07	0.02	0.07

** $P<0.01$, *** $P<0.001$

Table S2

Table of two-way ANOVA performed on arcsine-transformed Ki67-positive cell rates, with culture period and EGF treatment as two factors.

	F-value
	**Ki67 positive**
**Culture period**	33.350 ***
**EGF**	10.066 **
**Culture period × EGF**	6.473 **

**P < 0.01, ***P < 0.001

Table S3

Differentially expressed genes (DEGs) in the UG-LIKE group compared with the CYST group (an FDR-adjusted *P* < 0.05, Log2(FC) > 1 or < -1, and TPM > 1).

Name	Identifier	Max group means	Log fold change	FDR p-value
IGFBP3	ENSBTAG00000003994	459.26	4.65	8.77E-07
SPRR3	ENSBTAG00000013507	1,717.39	3.68	3.91E-06
MELTF	ENSBTAG00000002998	50.29	3.33	2.19E-16
ADSS1	ENSBTAG00000017616	32.32	3.3	1.26E-09
gene:ENSBTAG00000053794	ENSBTAG00000053794	23.34	3.18	3.00E-07
CMAH	ENSBTAG00000003892	66.7	3.17	4.02E-14
gene:ENSBTAG00000052092	ENSBTAG00000052092	371.58	3.17	3.66E-05
ADAMTS1	ENSBTAG00000000706	82.83	3.12	1.27E-06
TNFSF15	ENSBTAG00000018069	94.29	3.11	1.87E-16
MUC13	ENSBTAG00000010382	202.58	3.02	9.32E-14
ADGRF1	ENSBTAG00000021609	107.42	3	2.26E-14
LPAR6	ENSBTAG00000006645	85.13	2.99	3.00E-07
BCL2L14	ENSBTAG00000047225	23.95	2.96	8.27E-07
COL21A1	ENSBTAG00000033515	65.84	2.88	1.39E-08
CRABP2	ENSBTAG00000005716	85.06	2.87	2.74E-07
CYP4F22	ENSBTAG00000059781	34.44	2.8	4.59E-05
FCGR2B	ENSBTAG00000021842	34.2	2.79	9.04E-07
MYO1A	ENSBTAG00000014655	88.35	2.78	3.05E-15
GNA14	ENSBTAG00000021127	62.48	2.78	1.84E-06
MSLN	ENSBTAG00000000177	1,369.84	2.78	8.04E-05
SPP1	ENSBTAG00000005260	256.47	2.75	2.66E-05
ALDH1A3	ENSBTAG00000009125	392.25	2.74	3.85E-05
EVPL	ENSBTAG00000010517	23.33	2.73	2.71E-06
RAB27B	ENSBTAG00000002788	30.69	2.69	2.62E-05
C1GALT1	ENSBTAG00000004995	89.81	2.63	6.14E-14
MMP13	ENSBTAG00000015059	2,975.76	2.6	2.20E-09
ST6GAL1	ENSBTAG00000010357	11.01	2.59	4.96E-03
S100A4	ENSBTAG00000019203	424.39	2.59	6.68E-11
TFF3	ENSBTAG00000021276	544.17	2.57	7.05E-07
TCHH	ENSBTAG00000053103	107.22	2.54	9.44E-08
PKIB	ENSBTAG00000004394	19.84	2.54	3.18E-04
UPK3B	ENSBTAG00000021430	34.56	2.49	2.16E-04
ENPP3	ENSBTAG00000020196	136.91	2.48	3.72E-10
HYAL1	ENSBTAG00000000483	29.9	2.46	1.74E-03
LYPD6B	ENSBTAG00000019683	22.93	2.43	1.62E-04
GPX3	ENSBTAG00000043553	152.99	2.43	2.32E-11

AREG	ENSBTAG00000018134	80.59	2.41	8.84E-07
FER1L6	ENSBTAG00000013537	33.07	2.4	1.14E-03
NMB	ENSBTAG00000058029	55.38	2.4	3.07E-03
ANGPTL4	ENSBTAG00000002473	146.25	2.39	1.76E-03
GALNT5	ENSBTAG00000017376	16.39	2.38	5.53E-04
PAQR6	ENSBTAG00000009434	131.79	2.38	2.25E-12
FAM3D	ENSBTAG00000023207	149.1	2.37	3.87E-07
FA2H	ENSBTAG00000012222	19.8	2.35	1.02E-03
LYPD3	ENSBTAG00000018077	64.7	2.33	7.20E-07
gene:ENSBTAG00000051412	ENSBTAG00000051412	44.01	2.31	2.32E-03
TCF7L2	ENSBTAG00000021574	8.41	2.26	2.10E-03
SESN3	ENSBTAG00000004034	8.5	2.22	4.15E-03
RAB3B	ENSBTAG00000076305	32.77	2.21	3.43E-03
CLDN23	ENSBTAG00000007051	16.92	2.21	3.93E-04
UPK1B	ENSBTAG00000009014	124.06	2.2	1.08E-03
PALMD	ENSBTAG00000017655	189.79	2.19	8.25E-10
CD109	ENSBTAG00000013222	61.23	2.18	3.47E-10
QSOX1	ENSBTAG00000014191	505	2.17	9.31E-06
gene:ENSBTAG00000025920	ENSBTAG00000025920	101.08	2.12	1.48E-08
SERPINB12	ENSBTAG00000035171	10.58	2.12	6.56E-04
TARS3	ENSBTAG00000007651	10.79	2.07	4.07E-03
PORCN	ENSBTAG00000009282	41.85	2.07	0.02
CCDC152	ENSBTAG00000033221	8.08	2.06	0.04
FXYD3	ENSBTAG00000063825	35.12	2.05	1.82E-03
S100A14	ENSBTAG00000021377	443.26	2.04	6.96E-04
LAMA3	ENSBTAG00000027181	439.47	2.04	2.62E-05
UPK3BL2	ENSBTAG00000020524	1,044.69	2.03	2.68E-04
TRIM29	ENSBTAG00000009419	6.08	2.01	0.02
TXNIP	ENSBTAG00000020060	646.74	1.99	9.93E-05
SCEL	ENSBTAG00000032821	132.3	1.98	1.02E-06
LRATD1	ENSBTAG00000004948	24.39	1.96	1.87E-05
RALGAPA2	ENSBTAG00000014178	8.92	1.95	1.38E-04
HACD4	ENSBTAG00000017741	47.66	1.94	3.55E-06
CIDEB	ENSBTAG00000010236	78.13	1.94	6.80E-05
BMF	ENSBTAG00000012180	30.21	1.94	2.30E-04
TCP11L2	ENSBTAG00000002127	14.99	1.93	2.84E-03
MISP	ENSBTAG00000020269	188.13	1.93	7.98E-06
SLC46A3	ENSBTAG00000059801	15.75	1.93	7.31E-04
CRIP1	ENSBTAG00000047229	1,683.32	1.92	3.30E-05
TMEM54	ENSBTAG00000008331	165.18	1.88	8.05E-09
TSPAN1	ENSBTAG00000013320	5,240.19	1.87	8.50E-04
RORC	ENSBTAG00000017405	31.75	1.86	3.79E-06

PIK3IP1	ENSBTAG00000010667	57	1.86	3.56E-04
C10H15orf48	ENSBTAG00000004558	112.59	1.85	3.35E-03
KCNN4	ENSBTAG00000004588	61.48	1.85	1.12E-04
ADHFE1	ENSBTAG00000004476	12.63	1.84	0.03
TRPV4	ENSBTAG00000000031	9.69	1.84	5.96E-03
ERO1A	ENSBTAG00000015716	326.6	1.83	0.02
PFKFB3	ENSBTAG00000008401	327.35	1.83	0.05
NDRG2	ENSBTAG00000000843	269.53	1.82	1.95E-03
TSLP	ENSBTAG00000060087	179.77	1.8	3.50E-03
SLC18B1	ENSBTAG00000015242	11.38	1.8	4.41E-03
LGALS4	ENSBTAG00000016312	332.23	1.8	2.21E-07
SERPINB7	ENSBTAG00000037773	195.2	1.79	1.64E-05
PDE8B	ENSBTAG00000020205	27.93	1.78	1.08E-03
VWA1	ENSBTAG00000021294	182.87	1.77	1.26E-05
HDAC11	ENSBTAG00000007208	10.34	1.75	0.02
VWA7	ENSBTAG00000005630	76.81	1.75	4.99E-07
NPDC1	ENSBTAG00000046140	69.96	1.74	2.65E-03
PMAIP1	ENSBTAG00000049851	65.87	1.74	5.78E-04
SYT17	ENSBTAG00000014229	17.68	1.74	5.89E-03
MUC1	ENSBTAG00000017104	501.7	1.73	6.78E-03
TFPI2	ENSBTAG00000015844	375.56	1.73	1.90E-03
RAI2	ENSBTAG00000039049	25.77	1.73	1.61E-04
FBXL21	ENSBTAG00000025182	8.78	1.72	0.03
AHR	ENSBTAG00000007746	70.2	1.71	1.12E-04
CSRP2	ENSBTAG00000013406	96.56	1.71	1.25E-05
MGAT3	ENSBTAG00000012503	9.48	1.71	0.04
RASSF6	ENSBTAG00000011935	51.27	1.69	3.82E-05
ECM1	ENSBTAG00000003806	86.89	1.68	4.73E-03
LAMB3	ENSBTAG00000016542	859.97	1.68	2.84E-03
KLHL17	ENSBTAG00000013813	16.81	1.67	0.02
SETD9	ENSBTAG00000013426	9.74	1.67	0.02
ADGRG2	ENSBTAG00000000576	11.03	1.66	8.51E-03
LY6G6E	ENSBTAG00000000582	291.29	1.65	1.22E-03
QPCT	ENSBTAG00000013923	23.03	1.64	0.03
TNFRSF21	ENSBTAG00000020054	114.06	1.64	4.40E-03
CIST1	ENSBTAG00000033886	356.03	1.63	2.00E-04
ABHD2	ENSBTAG00000019954	42.09	1.63	9.08E-06
ITGB6	ENSBTAG00000009080	288.19	1.62	4.41E-03
SMPDL3A	ENSBTAG00000021587	26.46	1.62	0.03
MXD1	ENSBTAG00000001042	43.1	1.62	1.12E-04
GSTP1	ENSBTAG00000003548	27.21	1.62	0.04
SPTSSB	ENSBTAG00000049223	319.3	1.61	7.10E-05

EPHB6	ENSBTAG00000015510	31.2	1.61	0.04
ATOSB	ENSBTAG00000011394	42.43	1.61	4.68E-03
ITPRID2	ENSBTAG00000000937	12.51	1.59	0.03
SIRPA	ENSBTAG00000007213	18.08	1.58	1.37E-03
F2R	ENSBTAG00000020199	49.2	1.55	0.03
LGALS3	ENSBTAG00000002326	1,563.63	1.54	6.18E-03
MLKL	ENSBTAG00000012216	20.74	1.54	8.22E-03
PARM1	ENSBTAG00000015919	43.31	1.52	3.15E-03
PLCD1	ENSBTAG00000037726	76.78	1.52	6.95E-05
TFPI	ENSBTAG00000049919	136.28	1.51	9.93E-05
FADS6	ENSBTAG00000017242	26.97	1.51	5.05E-04
ID3	ENSBTAG00000030425	232.18	1.5	3.88E-03
CCND2	ENSBTAG00000016649	47.29	1.5	0.01
GCNT3	ENSBTAG00000009443	30.08	1.5	0.01
BLNK	ENSBTAG00000021358	73.66	1.5	5.08E-03
BTC	ENSBTAG00000004237	9.93	1.47	0.02
B3GNT3	ENSBTAG00000046750	204.14	1.47	6.75E-05
PLCD3	ENSBTAG00000006052	20.44	1.47	6.11E-03
CYP1A1	ENSBTAG00000001021	65.55	1.47	6.76E-03
ORAI2	ENSBTAG00000012050	49.21	1.47	0.01
PLEKHG6	ENSBTAG00000004208	29.82	1.46	3.99E-03
FGFBP1	ENSBTAG00000031497	400.16	1.46	0.02
VMAC	ENSBTAG00000006067	23.92	1.46	0.03
B4GALNT2	ENSBTAG00000009864	110.64	1.46	6.11E-03
PLXNA3	ENSBTAG00000008552	10.78	1.46	5.16E-03
ELAPOR1	ENSBTAG00000001136	474.14	1.45	0.01
TMPRSS6	ENSBTAG00000032152	61.83	1.45	6.61E-03
CAST	ENSBTAG00000000874	246.81	1.45	2.52E-03
DSC2	ENSBTAG00000040584	224.89	1.45	4.96E-03
RBMS2	ENSBTAG00000017830	40.07	1.44	9.85E-04
CAMK2D	ENSBTAG00000014463	22.18	1.44	6.66E-04
ATP6V1B1	ENSBTAG00000010620	50.87	1.43	5.48E-03
TRPM4	ENSBTAG00000006139	23.2	1.43	3.25E-03
ENC1	ENSBTAG00000026369	31.97	1.43	1.54E-03
TNFSF9	ENSBTAG00000046266	31.26	1.42	9.11E-03
GSN	ENSBTAG00000019915	894.9	1.42	4.22E-03
HOMER2	ENSBTAG00000017715	83.5	1.42	3.77E-04
IPMK	ENSBTAG00000011197	17.39	1.42	3.36E-03
PPP2R5A	ENSBTAG00000000754	105.4	1.4	2.68E-04
ITPR3	ENSBTAG00000013245	6.68	1.39	0.05
ATOSA	ENSBTAG00000024958	14.46	1.38	8.70E-03
ECI1	ENSBTAG00000009965	143.95	1.38	0.01

ITM2B	ENSBTAG00000003109	1,562.80	1.37	5.60E-03
CA5B	ENSBTAG00000020418	34.96	1.37	3.71E-03
CGNL1	ENSBTAG00000008575	60.88	1.36	2.34E-03
CD44	ENSBTAG00000011578	171.28	1.36	8.49E-04
BDH1	ENSBTAG00000000448	209.49	1.35	0.02
TCIM	ENSBTAG00000033304	145.34	1.35	0.04
CRYBG2	ENSBTAG00000005629	21	1.34	3.53E-03
TAOK3	ENSBTAG00000012550	9.87	1.34	0.04
SELENOP	ENSBTAG00000054085	150.34	1.34	0.02
NAALADL2	ENSBTAG00000048493	18.5	1.33	8.22E-03
DDAH2	ENSBTAG00000013530	232.19	1.33	3.82E-03
RHOBTB1	ENSBTAG00000019164	77.95	1.33	3.09E-04
SIAE	ENSBTAG00000009412	37.04	1.33	1.69E-03
SLC4A4	ENSBTAG00000002348	22.65	1.32	4.68E-03
MIER3	ENSBTAG00000014248	36.43	1.31	1.12E-03
MUC20	ENSBTAG00000032819	842.02	1.3	0.03
GIPC2	ENSBTAG00000056556	84.7	1.3	3.40E-03
STAT6	ENSBTAG00000006335	112.52	1.3	9.28E-04
RDH10	ENSBTAG00000020143	90.32	1.3	2.33E-03
SLC27A5	ENSBTAG00000015164	47.25	1.3	0.01
TUBB4A	ENSBTAG00000021013	124.52	1.29	4.84E-04
TMEM229B	ENSBTAG00000012900	21.32	1.29	4.19E-03
ZNF750	ENSBTAG00000015406	58.15	1.29	7.75E-03
EPS8L3	ENSBTAG00000001845	62.54	1.28	5.89E-03
C18H19orf33	ENSBTAG00000063903	280.64	1.28	3.49E-03
SH3D19	ENSBTAG00000016813	11.12	1.27	0.02
BIN1	ENSBTAG00000019177	254.01	1.26	3.07E-03
CDA	ENSBTAG00000068005	101.28	1.26	0.01
STBD1	ENSBTAG00000010389	112.66	1.26	1.30E-03
SLC4A7	ENSBTAG00000018227	10.39	1.26	0.01
COQ8A	ENSBTAG00000021880	30.97	1.25	0.04
CTSD	ENSBTAG00000007622	302.08	1.25	8.72E-03
TNFSF10	ENSBTAG00000057675	8.98	1.24	0.04
PLD1	ENSBTAG00000017490	18.75	1.24	0.05
FGD3	ENSBTAG00000006939	54.34	1.23	3.32E-03
LRP1	ENSBTAG00000010830	20.8	1.22	7.75E-03
C6H4orf19	ENSBTAG00000006643	55.3	1.22	0.02
TMEM116	ENSBTAG00000006659	19.4	1.22	9.25E-03
TMEM164	ENSBTAG00000037413	22.55	1.22	0.03
RIT1	ENSBTAG00000000105	192.63	1.21	0.02
REPS1	ENSBTAG00000012299	39.91	1.21	0.03
RCBTB2	ENSBTAG00000006647	61.49	1.21	1.13E-03

FBXO32	ENSBTAG00000016194	26.54	1.21	0.03
CAPN5	ENSBTAG00000005034	63.93	1.21	0.02
CKMT1A	ENSBTAG00000007502	1,300.85	1.21	0.02
MBOAT1	ENSBTAG00000016519	30.07	1.21	0.02
CD247	ENSBTAG00000012700	46.49	1.2	0.03
gene:ENSBTAG00000051567	ENSBTAG00000051567	100.31	1.2	0.03
PHYHD1	ENSBTAG00000038831	46.79	1.2	0.03
NNAT	ENSBTAG00000003212	171.53	1.2	4.46E-03
SELENOW	ENSBTAG00000066361	179.69	1.2	7.73E-03
TMEM106A	ENSBTAG00000006801	54.84	1.2	0.05
TMEM86A	ENSBTAG00000048059	155.93	1.2	0.04
CCDC28A	ENSBTAG00000015516	90.81	1.19	0.03
FAM177B	ENSBTAG00000049754	40.32	1.19	0.04
RASAL1	ENSBTAG00000016739	45.46	1.19	0.04
PAQR8	ENSBTAG00000025494	60.29	1.19	2.36E-03
PRKCD	ENSBTAG00000008719	59.6	1.17	9.70E-03
gene:ENSBTAG00000005448	ENSBTAG00000005448	198.17	1.16	7.52E-03
MDFIC	ENSBTAG00000021543	33.9	1.15	0.05
EPS8	ENSBTAG00000000369	41.07	1.15	0.02
CCNI	ENSBTAG00000000599	198.84	1.15	6.12E-03
HPCAL1	ENSBTAG00000004259	46.29	1.15	0.02
SLMAP	ENSBTAG00000021164	53.03	1.15	6.51E-03
MST1R	ENSBTAG00000015046	17.32	1.15	0.04
C1H3orf52	ENSBTAG00000016554	51.79	1.14	0.03
ACAD10	ENSBTAG00000001164	19.16	1.14	0.04
B3GNT8	ENSBTAG00000059912	10.66	1.14	0.02
CUL7	ENSBTAG00000012749	14.09	1.14	0.04
AHNAK	ENSBTAG00000013468	107.71	1.14	0.04
RAB25	ENSBTAG00000018914	279.63	1.13	4.40E-03
CALCOCO1	ENSBTAG00000015016	118.08	1.13	0.01
ANTXR2	ENSBTAG00000014324	77	1.13	0.02
UCP2	ENSBTAG00000003692	274.32	1.13	3.32E-03
KIAA0513	ENSBTAG00000020835	15.49	1.13	0.03
ITGAV	ENSBTAG00000019929	107.71	1.12	6.33E-03
CPNE8	ENSBTAG00000020914	25.98	1.1	0.03
CLTB	ENSBTAG00000010740	208.57	1.1	3.94E-03
PJA1	ENSBTAG00000021026	88.25	1.1	0.01
GRN	ENSBTAG00000018823	1,093.64	1.09	0.04
MAN2A2	ENSBTAG00000018905	19.33	1.09	0.03
B3GALT4	ENSBTAG00000025669	28.3	1.09	0.05
MYO5B	ENSBTAG00000019455	35.66	1.09	9.14E-03
NECTIN4	ENSBTAG00000017877	49.93	1.08	8.28E-03

FAM234A	ENSBTAG00000016571	283.04	1.08	0.04
YPEL3	ENSBTAG00000009047	114.87	1.08	6.12E-03
SLK	ENSBTAG00000001017	17.99	1.08	0.05
SLC36A4	ENSBTAG00000008530	15.5	1.08	0.04
POLD4	ENSBTAG00000017951	126.39	1.08	0.03
EFNB1	ENSBTAG00000015801	34.38	1.08	0.04
PBXIP1	ENSBTAG00000004552	74.34	1.07	9.97E-03
KLF3	ENSBTAG00000017488	44.54	1.07	0.04
LURAP1L	ENSBTAG00000010431	112.53	1.07	0.02
MTMR11	ENSBTAG00000012496	48.3	1.06	0.02
P2RY2	ENSBTAG00000062155	7.88	1.06	0.04
SLC37A1	ENSBTAG00000021286	92.2	1.05	6.02E-03
S100A16	ENSBTAG00000014204	276.33	1.05	0.05
TST	ENSBTAG00000030650	78.21	1.05	0.05
TET2	ENSBTAG00000017682	11.28	1.04	0.05
MACC1	ENSBTAG00000003751	27.47	1.03	0.03
TCAF1	ENSBTAG00000002554	50.26	1.03	0.02
ARSA	ENSBTAG00000016053	132.36	1.03	0.02
MSX1	ENSBTAG00000010875	311.04	1.03	0.04
ENTPD2	ENSBTAG00000048125	87.64	1.03	0.04
ELF3	ENSBTAG00000008756	169.79	1.03	0.04
KLK10	ENSBTAG00000015129	82.87	1.03	0.04
NPEPPS	ENSBTAG00000011435	138.69	1.03	0.04
REEP3	ENSBTAG00000019755	127.23	1.03	0.02
FBXO25	ENSBTAG00000007039	41.03	1.02	0.04
CREG1	ENSBTAG00000008931	137.05	1.02	0.04
YPEL5	ENSBTAG00000023744	201.53	1.02	0.02
ATP9A	ENSBTAG00000007962	57.35	1.02	0.01
MR1	ENSBTAG00000009924	72.95	1.02	0.02
FXYD5	ENSBTAG00000031441	208.48	1.02	0.03
SPHK1	ENSBTAG00000008507	32.05	1.02	0.04
MARCKSL1	ENSBTAG00000046862	107.01	1.01	0.02
TCTA	ENSBTAG00000022632	112.7	1	0.03
GCA	ENSBTAG00000018446	115.8	-1	0.04
GRWD1	ENSBTAG00000000080	59.03	-1	0.04
DDX39A	ENSBTAG00000021820	218.22	-1.01	0.03
DUT	ENSBTAG00000011998	114.71	-1.01	0.03
CRIM1	ENSBTAG00000009020	78.77	-1.01	0.03
GTPBP4	ENSBTAG00000014917	91.42	-1.01	0.02
NUP85	ENSBTAG00000016126	77.43	-1.01	0.02
ITGA5	ENSBTAG00000013745	70.37	-1.02	0.04
RPIA	ENSBTAG00000002866	74.15	-1.02	0.03

RMC1	ENSBTAG00000015190	61.61	-1.03	0.02
ADK	ENSBTAG00000011072	86.41	-1.03	0.02
POLA2	ENSBTAG00000011455	28.36	-1.03	0.03
TMLHE	ENSBTAG00000011648	42.84	-1.03	0.05
GET1	ENSBTAG00000013629	99.3	-1.04	7.37E-03
NCAPD3	ENSBTAG00000002260	16.33	-1.04	0.03
GFM2	ENSBTAG00000015519	38.64	-1.04	0.04
ACAD9	ENSBTAG00000003242	41.18	-1.04	0.04
ZWINT	ENSBTAG00000002655	66.93	-1.04	0.03
MAK16	ENSBTAG00000033504	90.76	-1.04	0.01
FXN	ENSBTAG00000001306	16.7	-1.05	0.02
TPX2	ENSBTAG00000018775	54.49	-1.05	0.05
POLR1G	ENSBTAG00000023601	51.51	-1.05	0.04
NME1	ENSBTAG00000004651	315.39	-1.05	8.02E-03
ECT2	ENSBTAG00000023814	63.02	-1.06	0.02
LAMB1	ENSBTAG00000011412	34.38	-1.06	0.02
NID2	ENSBTAG00000021945	19.24	-1.06	0.05
SELENOI	ENSBTAG00000007837	22.24	-1.06	0.02
POP5	ENSBTAG00000005385	168.68	-1.06	0.01
RAD51	ENSBTAG00000002918	48.58	-1.07	0.04
CENPS	ENSBTAG00000013531	88.39	-1.07	0.04
LRRC59	ENSBTAG00000006072	309.1	-1.07	0.04
NOLC1	ENSBTAG00000007435	110.45	-1.07	8.51E-03
TSNAX	ENSBTAG00000047434	167.42	-1.07	6.64E-03
OLA1_2	ENSBTAG00000052586	51.08	-1.07	0.03
CTPS1	ENSBTAG00000001626	39.18	-1.08	0.03
RRS1	ENSBTAG00000016716	66.75	-1.08	0.03
HSD11B1	ENSBTAG00000015086	64.05	-1.08	0.05
NQO1	ENSBTAG00000020632	91.55	-1.08	0.04
MANF	ENSBTAG00000060215	446.45	-1.08	0.01
IMP4	ENSBTAG00000003225	49.23	-1.09	0.01
C29H11orf98	ENSBTAG00000010465	42.94	-1.09	0.03
BCS1L	ENSBTAG00000003813	65.44	-1.1	0.02
NOP56	ENSBTAG00000018812	315.32	-1.1	0.01
PPA1	ENSBTAG00000007836	142.88	-1.1	6.70E-03
ESPL1	ENSBTAG00000008934	15.04	-1.11	0.05
OAT	ENSBTAG00000006928	177.15	-1.11	0.01
NXT2	ENSBTAG00000020739	97.4	-1.11	0.01
MRPL15	ENSBTAG00000001174	130.53	-1.12	4.68E-03
CTSH	ENSBTAG00000010992	202.14	-1.12	0.02
gene:ENSBTAG00000078356	ENSBTAG00000078356	197.89	-1.12	0.02
SMC2	ENSBTAG00000008772	27.28	-1.13	0.04

CYCS_2	ENSBTAG00000063185	40.16	-1.13	0.01
SAE1	ENSBTAG00000002676	277.99	-1.13	4.68E-03
THOC1	ENSBTAG00000019215	40.61	-1.13	0.02
TMEM126A	ENSBTAG00000000451	61.22	-1.13	0.05
EMG1	ENSBTAG00000013126	97.33	-1.14	0.01
HMGN2	ENSBTAG00000056502	1,870.00	-1.15	0.02
IMPA2	ENSBTAG00000043951	33.64	-1.15	0.02
MBOAT2	ENSBTAG00000008160	42.16	-1.16	0.02
DKC1	ENSBTAG00000013045	172.78	-1.16	1.69E-03
SMC4	ENSBTAG00000005862	29.29	-1.17	6.11E-03
GMNN	ENSBTAG00000017329	241.42	-1.17	1.93E-03
KIF11	ENSBTAG00000009383	44.16	-1.17	0.03
RRP1B	ENSBTAG00000017418	21.5	-1.18	0.03
LTV1	ENSBTAG00000032163	53.01	-1.18	0.01
DHODH	ENSBTAG00000019887	42.19	-1.18	0.03
POLD2	ENSBTAG00000012241	73.48	-1.19	7.19E-03
KIF23	ENSBTAG00000009983	46.44	-1.19	0.05
MTHFD1	ENSBTAG00000009641	147.54	-1.19	4.58E-03
PCNA	ENSBTAG00000006065	244.87	-1.19	4.12E-03
BDKRB2	ENSBTAG00000021717	29.55	-1.19	0.02
DYNC2I1	ENSBTAG00000024157	97.16	-1.2	1.98E-03
SRSF7	ENSBTAG00000014891	446.69	-1.2	3.88E-03
PTCD3	ENSBTAG00000006482	46.02	-1.2	0.01
GUCY1B1	ENSBTAG00000003840	47.66	-1.2	9.94E-03
BRI3BP	ENSBTAG00000001543	47.21	-1.2	0.01
C18H19orf48	ENSBTAG00000011079	63.67	-1.2	0.01
BUB1B	ENSBTAG00000007237	32.91	-1.21	0.05
ACTG2	ENSBTAG00000015441	69.64	-1.21	0.04
ACSS1	ENSBTAG00000004281	46.46	-1.21	0.01
PUS7	ENSBTAG00000007743	27.81	-1.22	3.94E-03
SDF2L1	ENSBTAG00000000067	77.8	-1.22	0.01
B9D1	ENSBTAG00000017790	31.49	-1.22	0.03
RUVBL1	ENSBTAG00000020998	188.21	-1.22	7.82E-04
TFRC	ENSBTAG00000032719	191.78	-1.23	2.23E-03
WDR77	ENSBTAG00000014102	72.65	-1.23	7.73E-03
CGRRF1	ENSBTAG00000017141	27.03	-1.23	0.02
RCN1	ENSBTAG00000047362	310.41	-1.23	0.03
NEK2	ENSBTAG00000009618	66.92	-1.23	0.01
TOMM40	ENSBTAG00000001781	64.64	-1.23	9.04E-03
SPC25	ENSBTAG00000010048	39.84	-1.24	6.31E-03
CDCA2	ENSBTAG00000002756	45.44	-1.24	0.02
MYBBP1A	ENSBTAG00000007430	75.9	-1.24	1.34E-03

FAP	ENSBTAG00000008140	36.65	-1.25	0.04
PSAT1	ENSBTAG00000013960	461.32	-1.25	0.04
FAM83D	ENSBTAG00000000660	39.62	-1.25	9.14E-03
CDC45	ENSBTAG00000004286	22.1	-1.25	0.03
OXCT1	ENSBTAG00000033186	52.02	-1.25	3.50E-03
HMGB2	ENSBTAG00000015101	446.05	-1.27	3.80E-03
MTHFD2	ENSBTAG00000004881	40.86	-1.27	3.46E-03
RRM1	ENSBTAG00000013111	437.78	-1.27	0.01
EEF1E1	ENSBTAG00000002534	74.11	-1.27	4.02E-03
SLC35E3	ENSBTAG00000031919	26.09	-1.28	4.96E-03
PRMT5	ENSBTAG00000010890	99.58	-1.28	9.19E-04
BIRC5	ENSBTAG00000013573	186.87	-1.28	0.02
CTSC	ENSBTAG00000011100	25.96	-1.28	5.84E-03
ALG3	ENSBTAG00000005652	42.37	-1.29	0.04
SGO1	ENSBTAG00000000752	23.92	-1.29	0.04
CDCA8	ENSBTAG00000014326	53.3	-1.29	0.01
NAT10	ENSBTAG00000016747	42.35	-1.29	2.84E-03
BRCA1	ENSBTAG00000022520	20.55	-1.29	0.01
DLGAP5	ENSBTAG00000002331	33.6	-1.3	0.01
MCM10	ENSBTAG00000016406	39.3	-1.3	5.25E-03
AURKA	ENSBTAG00000013009	44.53	-1.3	0.01
MLLT11	ENSBTAG00000015369	54.79	-1.31	0.03
CHAF1A	ENSBTAG00000008181	25.53	-1.31	9.49E-03
SLC5A6	ENSBTAG00000006832	56.5	-1.31	5.13E-03
SERPINH1	ENSBTAG00000001027	52.18	-1.31	8.97E-03
CDCA3	ENSBTAG00000019777	58.96	-1.32	8.56E-03
TMEM123	ENSBTAG00000049233	235.43	-1.32	0.02
SPAG5	ENSBTAG00000013100	48.17	-1.32	0.02
PFAS	ENSBTAG00000031509	39.8	-1.32	1.13E-03
PRPS1	ENSBTAG00000019703	41.15	-1.32	3.90E-03
PLPP3	ENSBTAG00000011640	148.11	-1.33	1.17E-03
CDC20	ENSBTAG00000009819	135.25	-1.33	1.05E-03
gene:ENSBTAG00000032914	ENSBTAG00000032914	52.94	-1.33	1.16E-04
CWH43	ENSBTAG00000021347	317.6	-1.33	0.01
MMP3	ENSBTAG00000037768	3,851.99	-1.33	0.02
AGPAT5	ENSBTAG00000004922	17.88	-1.33	0.04
TPM2	ENSBTAG00000011424	51.7	-1.34	0.03
BRCA2	ENSBTAG00000000988	5.9	-1.34	0.03
CRELD2	ENSBTAG00000047801	105.54	-1.35	1.87E-03
MMP9	ENSBTAG00000020676	216.56	-1.35	0.02
TRPA1	ENSBTAG00000002062	49.47	-1.35	2.28E-03
CAD	ENSBTAG00000017894	21.67	-1.36	5.66E-03

FANCI	ENSBTAG00000009097	29.82	-1.36	4.26E-03
FEN1	ENSBTAG00000000064	36.46	-1.36	7.82E-03
RFC4	ENSBTAG00000014727	53.42	-1.37	0.01
OLFML3	ENSBTAG00000011327	73.84	-1.37	4.71E-03
GCNT1	ENSBTAG00000012757	6.13	-1.37	0.03
EFR3B	ENSBTAG00000007888	27.62	-1.37	0.03
GJB1	ENSBTAG00000020512	13.69	-1.37	0.02
SFXN1	ENSBTAG00000014536	33.98	-1.38	5.40E-03
CPXM1	ENSBTAG00000011458	23.8	-1.38	0.04
WDHD1	ENSBTAG00000019120	25.48	-1.39	1.74E-03
UBE2C	ENSBTAG00000016746	123.11	-1.39	6.04E-03
RMI2	ENSBTAG00000026375	43.53	-1.39	0.03
ACSL4	ENSBTAG00000018986	357.02	-1.39	9.70E-03
FIGNL1	ENSBTAG00000001500	7.13	-1.4	0.04
DDX11	ENSBTAG00000007093	13.5	-1.4	0.02
BCKDHB	ENSBTAG00000012096	40.43	-1.4	4.96E-03
HELLS	ENSBTAG00000005979	24.58	-1.41	8.22E-03
CXCL8	ENSBTAG00000019716	47.5	-1.42	0.03
KNL1	ENSBTAG00000053398	12.88	-1.42	0.03
NUSAP1	ENSBTAG00000010774	100.95	-1.42	7.07E-03
SLCO4A1	ENSBTAG00000016388	14.86	-1.42	0.04
SPDL1	ENSBTAG00000008180	34.06	-1.42	3.72E-03
SNRNP25	ENSBTAG00000019804	74.1	-1.42	0.04
SLC5A5	ENSBTAG00000015830	47.78	-1.43	1.54E-03
BRIP1	ENSBTAG00000012068	8.12	-1.43	0.05
KPNA2	ENSBTAG00000012225	443.91	-1.43	4.75E-03
LMNB1	ENSBTAG00000002882	112.01	-1.44	0.01
TOP2A	ENSBTAG00000019262	116.48	-1.44	0.02
RAD51AP1	ENSBTAG00000040065	33.37	-1.45	0.02
KIF22	ENSBTAG00000013669	103.31	-1.45	6.07E-04
MELK	ENSBTAG00000021686	39.95	-1.46	1.17E-03
CENPN	ENSBTAG00000011635	53	-1.46	2.65E-03
SFXN3	ENSBTAG00000005015	9.42	-1.46	0.05
CCNE2	ENSBTAG00000004906	20.47	-1.47	0.04
VRK1	ENSBTAG00000014230	22.4	-1.47	6.66E-04
TCF19	ENSBTAG00000014435	25.48	-1.47	0.03
SPC24	ENSBTAG00000006185	85.09	-1.48	0.01
CCNB1	ENSBTAG00000014239	241.66	-1.48	1.19E-03
ERCC6L	ENSBTAG00000005607	12.15	-1.48	0.03
CCNA2	ENSBTAG00000004943	75.85	-1.49	0.01
MCM6	ENSBTAG00000015172	108.27	-1.5	1.60E-04
ACP5	ENSBTAG00000004826	256.03	-1.5	0.03

TAGLN	ENSBTAG00000007196	92.05	-1.5	8.97E-03
NCAPG2	ENSBTAG00000016131	23.32	-1.51	2.32E-03
GNB5	ENSBTAG00000003762	10.26	-1.52	8.35E-03
PDXK	ENSBTAG00000074909	43.51	-1.53	0.03
ORC5	ENSBTAG00000040058	21.64	-1.53	0.02
CXCL14	ENSBTAG00000006694	128.62	-1.53	0.02
MCM4	ENSBTAG00000017021	53.07	-1.53	1.31E-03
CALD1	ENSBTAG00000013953	42.29	-1.54	2.62E-05
CENPK	ENSBTAG00000044175	38.04	-1.54	0.01
DIPK1A	ENSBTAG00000002028	18.92	-1.55	0.04
BCAT1	ENSBTAG00000013825	26.51	-1.55	9.37E-04
TACC3	ENSBTAG00000011044	48.62	-1.55	4.16E-04
ACSL6	ENSBTAG00000019708	7.1	-1.55	0.02
CENPU	ENSBTAG00000078062	20.74	-1.55	0.03
FBLN5	ENSBTAG00000018123	8.87	-1.56	0.04
INHBB	ENSBTAG00000049058	13.76	-1.57	0.02
PTTG1	ENSBTAG00000012184	125.36	-1.57	1.06E-03
PBK	ENSBTAG00000021069	68.99	-1.57	2.66E-03
NCAPH	ENSBTAG00000012925	20.52	-1.57	0.01
RPP40	ENSBTAG00000003332	7.83	-1.57	0.02
CHAF1B	ENSBTAG00000011880	32	-1.58	0.02
DIRAS3	ENSBTAG00000012182	26.18	-1.58	6.53E-03
SLC26A7	ENSBTAG00000032301	56.06	-1.58	5.24E-03
RNF2	ENSBTAG00000000023	50.43	-1.58	1.17E-03
SLC25A5	ENSBTAG00000046037	562.15	-1.58	2.69E-04
GSTM5	ENSBTAG00000037673	90.02	-1.59	1.07E-03
KIF2C	ENSBTAG00000015280	49.02	-1.59	3.26E-03
CXCL2	ENSBTAG00000027513	43.69	-1.59	0.02
SHCBP1	ENSBTAG00000033441	45.53	-1.59	1.92E-03
ME1	ENSBTAG00000005681	52.37	-1.6	5.60E-03
KIFC1	ENSBTAG00000001631	23.93	-1.6	2.33E-03
CTTNBP2	ENSBTAG00000021469	32.22	-1.61	2.38E-04
SPARCL1	ENSBTAG00000004094	59.01	-1.61	2.70E-03
PODXL	ENSBTAG00000010452	23.94	-1.62	0.02
FBXO5	ENSBTAG00000021193	12.44	-1.62	0.03
AURKB	ENSBTAG00000001717	68.54	-1.62	2.78E-04
ASF1B	ENSBTAG00000004085	86.32	-1.63	2.15E-04
OIP5	ENSBTAG00000010766	27.88	-1.63	0.03
SFXN2	ENSBTAG00000004321	24.1	-1.63	5.23E-03
GINS4	ENSBTAG00000017133	9.43	-1.63	0.04
P3H1	ENSBTAG00000017382	52.92	-1.64	2.65E-05
DTYMK	ENSBTAG00000015408	102.27	-1.64	2.42E-04

SLC1A1	ENSBTAG00000019125	131.47	-1.64	1.06E-05
SLC25A48	ENSBTAG00000018287	28.72	-1.65	0.02
PALM2	ENSBTAG00000014471	9.93	-1.65	0.03
SURF2	ENSBTAG00000000046	30.23	-1.65	0.02
MCM2	ENSBTAG00000014380	45.51	-1.66	1.15E-04
STXBP1	ENSBTAG00000003525	28.2	-1.67	4.56E-04
COL8A1	ENSBTAG00000013662	7.05	-1.68	0.02
PLK1	ENSBTAG00000014453	25.91	-1.68	5.05E-04
P2RY1	ENSBTAG00000001465	5.1	-1.69	0.02
E2F8	ENSBTAG00000017446	15.31	-1.69	0.02
SOD3	ENSBTAG00000013980	21.08	-1.7	9.83E-04
TTK	ENSBTAG00000005456	25.39	-1.7	4.75E-03
PCLAF	ENSBTAG00000039462	114.96	-1.71	1.83E-05
MCM3	ENSBTAG00000010721	112.65	-1.71	3.85E-05
CGREF1	ENSBTAG00000051104	21.63	-1.73	0.02
CD40	ENSBTAG00000020736	19.07	-1.73	0.04
PDPN	ENSBTAG00000001788	282.44	-1.73	9.90E-04
NCAPG	ENSBTAG00000021582	55.46	-1.74	1.16E-04
CRYAB	ENSBTAG00000000434	650.27	-1.74	5.05E-04
CXCR4	ENSBTAG00000001060	47.72	-1.75	1.17E-04
TRIP13	ENSBTAG00000006972	49.69	-1.77	6.13E-05
PRR15	ENSBTAG00000045567	52.58	-1.78	5.24E-03
ENPP1	ENSBTAG00000021830	16.03	-1.8	0.03
MTFR2	ENSBTAG00000007799	36.24	-1.8	3.38E-04
DTL	ENSBTAG00000018142	21.13	-1.8	4.73E-03
FIRRM	ENSBTAG00000014793	13.33	-1.81	1.93E-03
PPM1K	ENSBTAG00000005754	5.8	-1.82	0.04
UCHL1	ENSBTAG00000005078	20.06	-1.85	0.02
ESCO2	ENSBTAG00000006551	47.14	-1.85	1.08E-03
RNF180	ENSBTAG00000043992	7.05	-1.85	0.01
SLC25A20	ENSBTAG00000000191	9.43	-1.85	0.05
MND1	ENSBTAG00000000281	25.01	-1.86	0.01
CHST12	ENSBTAG00000020898	8.26	-1.86	0.01
MCM5	ENSBTAG00000015595	68.71	-1.87	4.82E-08
BEX3	ENSBTAG00000006026	14.86	-1.87	2.26E-03
MPC1	ENSBTAG00000027879	143.21	-1.88	6.84E-07
PALM3	ENSBTAG00000046744	7.01	-1.89	0.03
PINX1	ENSBTAG00000000500	22.24	-1.9	9.41E-03
RRM2	ENSBTAG00000008216	193.13	-1.92	1.51E-05
NIBAN1	ENSBTAG00000014548	28.08	-1.93	6.09E-04
RHBG	ENSBTAG00000012234	9.32	-1.94	0.03
STC1	ENSBTAG00000001687	277.52	-1.94	4.87E-03

DCDC2	ENSBTAG00000009798	166.15	-1.98	8.57E-09
ACKR4	ENSBTAG00000019577	15.16	-1.99	1.78E-03
RBP4	ENSBTAG00000000442	1,474.33	-2	1.51E-05
TMEM213	ENSBTAG00000001527	87.81	-2.01	4.33E-03
CREM	ENSBTAG00000016060	25.21	-2.04	7.19E-04
UHRF1	ENSBTAG00000002224	126.82	-2.06	7.52E-08
GPC6_2	ENSBTAG00000062823	8.61	-2.06	3.50E-04
SKA1	ENSBTAG00000018216	26.53	-2.06	2.33E-03
NEXN	ENSBTAG00000008921	6.32	-2.07	0.03
MIS18A	ENSBTAG00000075873	12.68	-2.08	1.51E-04
CRYGS	ENSBTAG00000003140	27.15	-2.08	0.03
FOSL1	ENSBTAG00000006194	14.9	-2.09	0.02
MOXD1	ENSBTAG00000019460	12.76	-2.1	0.03
CLDN8	ENSBTAG00000039820	53.74	-2.12	1.82E-04
SNAP91	ENSBTAG00000009355	33.82	-2.12	5.06E-07
NUP210	ENSBTAG00000003665	18.09	-2.13	2.08E-05
SLC29A1	ENSBTAG00000015131	43.64	-2.14	2.65E-08
gene:ENSBTAG00000038770	ENSBTAG00000038770	11.78	-2.16	0.01
GINS2	ENSBTAG00000044006	37.07	-2.18	3.73E-05
TMEM37	ENSBTAG00000026825	14.33	-2.24	0.02
INHBA	ENSBTAG00000056438	486.48	-2.25	3.03E-07
IL17RB	ENSBTAG00000011178	9.07	-2.25	5.44E-03
PHGDH	ENSBTAG00000039719	493.52	-2.26	1.27E-06
SLC39A14	ENSBTAG00000019225	11.68	-2.28	1.51E-05
MME	ENSBTAG00000002075	73.29	-2.29	5.00E-08
ORC1	ENSBTAG00000002719	17.54	-2.29	1.46E-04
CDC6	ENSBTAG00000010384	26.96	-2.3	2.59E-05
MAOB	ENSBTAG00000001288	39.63	-2.32	8.65E-06
gene:ENSBTAG00000004415	ENSBTAG00000004415	21.2	-2.36	2.18E-04
FOLR3	ENSBTAG00000071393	649.79	-2.37	6.11E-03
SEC11C	ENSBTAG00000011954	142.23	-2.39	1.80E-10
PROSER2	ENSBTAG00000039571	15.44	-2.45	8.97E-05
CSDC2	ENSBTAG00000004004	9.12	-2.48	9.01E-04
FBXO15	ENSBTAG00000009686	12.11	-2.52	3.75E-03
CLDN10	ENSBTAG00000003568	77.2	-2.56	5.20E-04
MAP2K6	ENSBTAG00000001609	90.84	-2.56	1.11E-06
CFI	ENSBTAG00000034501	53.03	-2.67	5.05E-04
MEP1B	ENSBTAG00000020391	72.86	-2.68	4.84E-04
ACMSD	ENSBTAG00000008039	13	-2.7	1.95E-03
gene:ENSBTAG00000021522	ENSBTAG00000021522	8.36	-2.72	7.63E-03
SLC11A2	ENSBTAG00000032902	257.47	-2.73	5.39E-10
gene:ENSBTAG00000050427	ENSBTAG00000050427	184.48	-2.83	2.58E-03

CTH	ENSBTAG00000014791	81.63	-2.86	1.73E-10
NPL	ENSBTAG00000002266	18.46	-2.89	2.87E-07
MGP	ENSBTAG00000012370	102.21	-2.91	1.53E-03
gene:ENSBTAG00000047990	ENSBTAG00000047990	162.01	-3.02	1.16E-03
IL6R	ENSBTAG00000018474	34.07	-3.11	1.83E-05
EDN1	ENSBTAG00000008096	155.01	-3.22	2.76E-19
FABP3	ENSBTAG00000016819	122.79	-3.24	2.25E-16
ANPEP	ENSBTAG00000016881	159.17	-3.3	3.14E-14
ELOVL2	ENSBTAG00000002497	54.44	-3.87	2.03E-26
GRP	ENSBTAG00000004796	42,262.78	-7.33	1.88E-73
gene:ENSBTAG00000007392	ENSBTAG00000007392	6,291.98	-7.5	1.00E-62

Table S4

Table of two-way ANOVA performed on arcsine- transformed formation rate of cysts, aggregates, and uterine gland-like structures, and logistic-transformed length of uterine gland-like structures, with collagen I supplementation and Y-27632 treatment as two factors.

	F-value			
	**Cysts**	**Aggregates**	**Uterine gland-like structures**	**Length**
**Y-27632 treatment**	1.999	1.200	2.216	27.337 ***
**collagen I supplementation**	0.001	0.163	0.200	26.867 ***
**Y-27632 × collagen I**	0.001	0.156	0.190	7.159 *

**P* < 0.05, ****P* < 0.001
