# Supplementary material for: Tubulogenesis of bovine uterine glands by epidermal growth factor and collagen I in 3D culture systems
Source: Biosci Rep. 2026 Jun 8;46(6):BSR20260010. doi: 10.1042/BSR20260010 (PMC13259824; doi:10.1042/BSR20260010)
Supplement: Supplementary Figures Video S1 [file BSR-2026-0010_supp1.pdf]

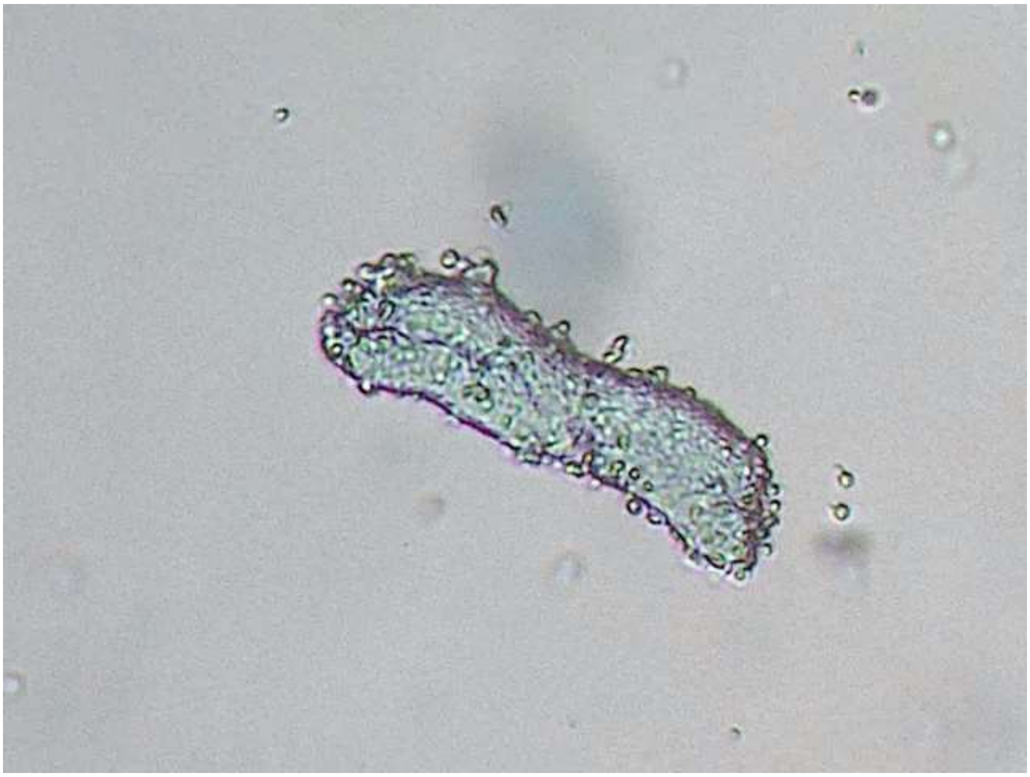

Video S1

Time-lapse imaging of bovine uterine gland-like structure formation in a 3D culture system. This video shows the morphological changes of 3D-cultured bovine uterine gland fragments in the presence of 5 ng/ml EGF. Images were captured every 20 min for a total duration of 5 days using a WSL-1800 CytoWatcher equipped with an ImageSaverT. The temperature was maintained at 38.5ºC in a humidified atmosphere containing 5% CO_2 in air.
